# Nanoionics Drastically Accelerating Mass Transfer at Elevated Temperatures over 750 °C

**DOI:** 10.1021/acsnano.5c17239

**Published:** 2026-02-12

**Authors:** Yun Chen, Cesar-Octavio Romo-De-La-Cruz, Fuming Jiang, Sergio Andres Paredes Navia, Xueyan Song

**Affiliations:** Department of Mechanical, Materials, and Aerospace Engineering, 5631 West Virginia University, Morgantown, West Virginia 26506, United States

**Keywords:** Nanoionics, Mass Transfer, Elevated Temperatures, Reversible Solid Oxide Cells, Conductivity, Interface, Atomic Layer Deposition

## Abstract

Nanoionics were previously considered thermally unstable and infeasible for devices operating above 500 °C. Here, we elucidate the design principle for establishing stable nanoionics from various oxides. We utilized reversible solid oxide cells (SOCs) as the test bed and implemented nanoionics using atomic layer deposition (ALD). We demonstrate a straightforward, interface-controlled, practical approach to render a conformal, ∼15 nm thick ALD film, which initially thermodynamically favors the formation of a solid solution with the substrate into surface nanoionics with single or double layers of nanograins with random crystal orientations. The nanoionics exhibited conductivity estimated to be 7 orders of magnitude higher than that of their bulk-scale counterpart. They demonstrated conformability with uniform grain sizes of ∼15 nm, even after electrochemical operation for ∼500 h at 750 °C and 1000 h at 850 °C. The thermal stability and conductivity of such nanoionics represent a conceptual and technological framework in nanoionics.

## Graphical abstract

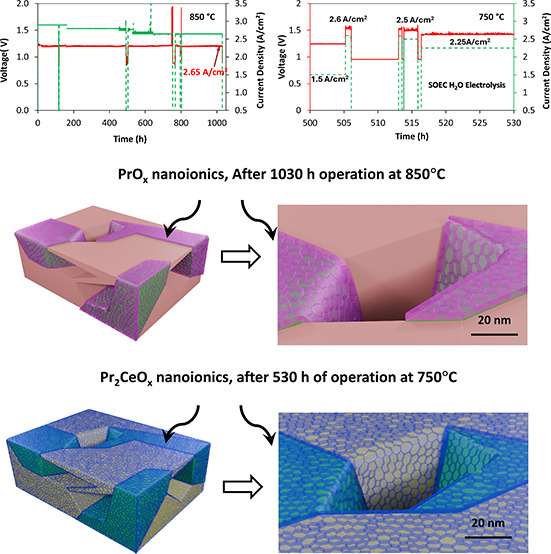

## Introduction

Ionic mass transfer and ionic conductivity are essential in electrochemical energy conversion, gas sensing, catalysis, and emerging iontronics.^1–5^ Nevertheless, unlike electronic conductivity, where orders of magnitude enhancements can be achieved by merely increasing the carrier concentration through doping, the use of dopants in enhancing ionic conductivity reaches a limit where a further increase in doping will impair ion mobility and reduce conductivity.^6^ Beyond this intragranular composition-optimized limit, the intergranular crystal imperfections, including the grain boundaries and the crystal interfaces, are creatively utilized to accelerate mass transfer and ionic conductivity through the space-charge effect of charge-carrier accumulation or the strain effect of reduced activation enthalpy for charge-carrier migration.^7–12^

In the field of oxide ceramics, although defect-facilitated nanoionics incorporating high-density grain boundaries and nanocomposites with heterogeneous interfaces have been actively pursued by the research community over the past few decades,^13,14^ they have barely been achieved at elevated temperatures of greater than 500 °C and fail to sustain reasonably long durations, such as several hundreds of hours.^15^ Defect-engineered nanostructured materials are thermally sensitive and can undergo agglomeration and rapid grain growth, reaching grain sizes exceeding 100 nm at temperatures above ∼400 °C.^16^ The elevated temperature, which is generally required for either the synthesis of oxides or their application, has limited the incorporation of nanoionics into practical devices. On the other hand, increasing ionic conductivity is essential for some critical high-temperature energy conversions, such as solid oxide fuel cells (SOFCs), which operate at ∼750 °C.^17,18^ The flexibility of SOFCs is demonstrated by their ability to generate electricity efficiently using various fuels, including natural gas (methane, CH_4_), ethane (C_2_H_6_), and propane (C_3_H_8_). Most uniquely, among the different fuel cells, including the Proton Exchange Membrane fuel cell, SOFC is the only type of fuel cell that can principally operate reversibly as solid oxide electrolysis cells (SOECs) to electrochemically split H_2_O and CO_2_, for solar fuel production and oxygen generation for astronauts’ life support, and for hydrogen and carbon monoxide (CO) for methane-based propellant on Mars, as well as scalable long-duration energy storage.^19–21^

Nevertheless, switching from SOFC to SOEC for hydrogen, oxygen, and carbon monoxide production, with the desired high electrolysis current density and high gas production rate, is not straightforward.^9,18^ The SOEC typically experiences severe degradation, including immediate catastrophic electrode delamination, primarily due to the lack of ionic conductivity in the constituent phases of the oxygen electrode. Enhancing the ionic conductivity of that from the SOFC is a necessity for enabling the SOEC. For the SOFC that has both materials set and manufacturing matured over the past decades, elevating ionic conductivity through implanting nanoionics into inherently functional state-of-the-art SOFCs could be one of the most straightforward approaches for revolutionizing SOEC technologies and ushering in a new era driven by artificial intelligence and space exploration. The latest scientific knowledge gained through the synthesis and processing of materials for stabilizing nanoionics is indispensable for providing the technological advancement needed in areas such as long-term energy storage and energy conversion, especially in devices that operate at elevated temperatures.

For the first time in the field of nanoionics, our work has previously demonstrated the atomic layer deposition (ALD) coating and implantation of nanoionics of ZrO_2_,^22^ CoO_*x*_,^23^ and MnCoO_*x*_ ^24^ on the internal surface of the air-electrode of SOFCs to simultaneously boost their peak power density and increase the durability over long-term electrochemical operation. In this study, we implant nanoionics into the internal surface of the air electrode of the as-fabricated, inherently functional SOFC to enable its reversibility as an SOEC by increasing the ionic conductivity of the air electrode and thereby preventing delamination during SOEC operation. Most importantly, we elucidate the design principle for establishing thermally stable nanoionics using various oxides. We demonstrate a straightforward, crystal-interface-controlled, practical approach to render the formation of the amorphous ALD film, which originally thermodynamically tends to form a solid solution with the substrate at elevated temperatures, into thermally stable, conformal, and uniform surface nanoionics.

## Results and Discussion

The SOFCs chosen for implanting nanoionics have composite air electrodes consisting of La_0_._8_Sr_0_._2_MnO_3_ (LSM), which is a pure electronic conductor lacking ionic conductivity and is balanced with the ionic conductor scandium-stabilized zirconium (SSZ) oxide. We performed ALD to coat conformal thin films, which are aimed at subsequently forming surface nanoionics on the internal surface of the porous air electrode, where electrochemical reactions occur. These surface nanoionics thus experience additional thermodynamic and kinetic driving forces from the electrochemical reactions, which could further assist in triggering thermally activated crystal grain growth at elevated temperatures.^25^ We applied an ALD coating of a minute amount of PrO_*x*_, which possesses electronic conductivity 4 orders of magnitude lower than that of LSM and ionic conductivity 3 orders of magnitude lower than that of SSZ at the bulk scale. Yet, PrO_*x*_ has proven to be an effective electrocatalyst due to its mixed valence state and high oxygen surface exchange.^26–29^

The as-deposited state of the uniform ALD layer of PrO_*x*_ is amorphous. A 15 nm thick indifferent coating is present on the LSM and SSZ, as shown in the transmission electron microscopy (TEM) image of Figure 1A. The SOFC with ALD coating on the LSM/SSZ was subjected to SOFC operation for 24 h and then switched to SOEC operation for an extended operation of 1030 h at 850 °C. After the electrolysis, the TEM image of the porous air-electrode, as shown in Figure 1A, displayed that a ∼15 nm thick PrO_*x*_ layer appears to diffuse into LSM and form a solid solution of (La_0.6_Sr_0.17_Pr_0.22_)­MnO_*x*_. By contrast, the PrO_*x*_ does not have solubility in SSZ and is immiscible with the SSZ. The PrO_*x*_ on SSZ forms single-layer conformal nanoionics with nanograins elongated ∼30 nm along the SSZ surface and ∼15 nm in thickness. As such, the ALD-coated PrO_*x*_ exhibited the bimodal nanostructure distribution on the internal surface of the LSM/SSZ substrate after 1030 h of electrolysis at 850 °C, as schematized in Figure 1C. The immiscibility between the ALD film and the substrate seems to influence the formation of the conformal PrO_*x*_ nanoionics on SSZ.

**Figure 1:**
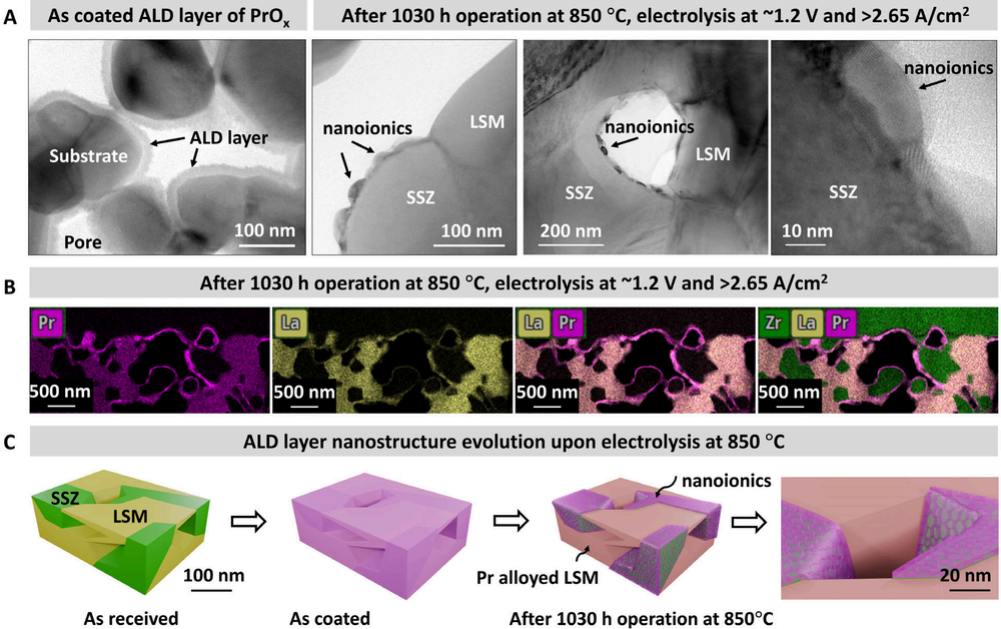
Bimodal structured porous electrode backbone and its internal surface. (A) TEM images of uniform as-deposited ALD coating of PrOx and the bimodal surface structure after electrolysis at 850 °C for 1030 h. (B) Scanning TEM (STEM) image of chemistry maps of different elements after 1030 h of electrolysis at 850 °C. (C) The schematic shows the bimodal surface structure and nanoionics that are present singly on SSZ.

To demonstrate the significance and versatility of forming stable nanoionics using examples with different miscibility with LSM, the CeO_*x*_,^30^ which is immiscible with LSM, was chosen as the subjacent layer for the superjacent PrO_*x*_. The ALD coating with a thickness of ∼15 nm was achieved through ALD coating of 3 nm subjacent CeO_*x*_ first, as shown in Figure 2A, followed by superjacent alternating PrO_*x*_ and CeO_*x*_. The cell with ALD coating of CeO_*x*_/PrO_*x*_ on the LSM/SSZ air electrode was subjected to SOFC operation for 24 h and then switched to SOEC operation for an extended period of over 530 h at 750 °C. The postoperation TEM images of the air electrode are shown in Figure 2A,B. The CeO_*x*_ appears to be alloyed with PrO_*x*_, forming the Pr_2_CeO_*x*_, which establishes conformal unimodal and dual-layered nanoionics on both LSM and YSZ, regardless of the substrate surface roughness and substrate grain orientation. The nanograins within the surface nanoionics are nonepitaxial with respect to the substrate and exhibit random crystal orientation. The interface between the Pr_2_CeO_*x*_ layer and the LSM grains is sharp at the atomic level, without any sign of interdiffusion between the coating layer and the substrate. Accordingly, we transitioned the surface coating layer of PrO_*x*_ from forming a solid solution to establishing stable surface nanoionics on LSM. We successfully rendered the formation of stable, unimodal surface nanoionics on both SSZ and LSM. The unimodal distribution of nanoionics on the LSM/SSZ substrate after 530 h of electrolysis at 750 °C is schematized in Figure 2C. The immiscibility between the substrate and the film has proven to be necessary for the formation of conformal surface nanoionics. The energy of the interface between the nanoionics and the substrate may have been a dominant factor in constraining the growth of the nanograins.

**Figure 2:**
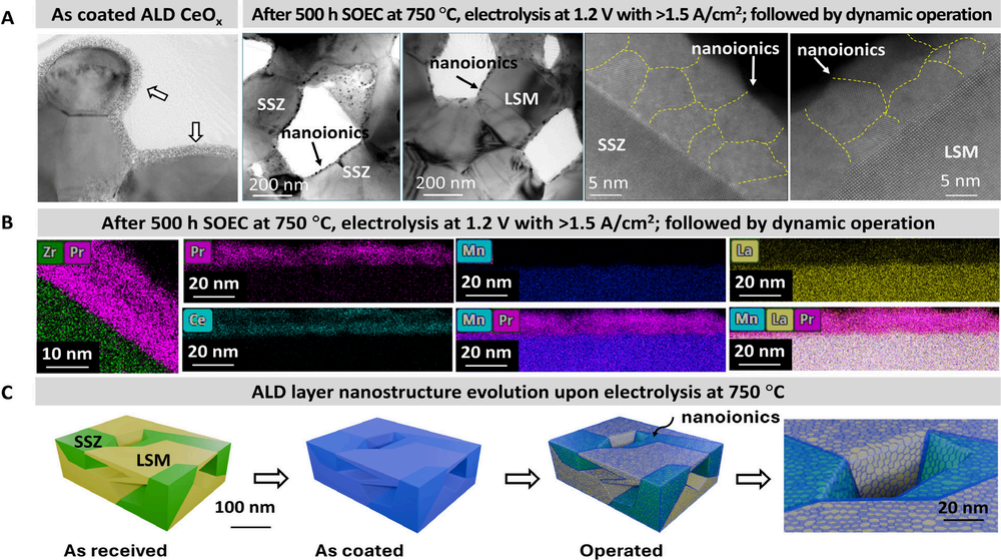
Unimodal conformal nanoionics on the porous backbone internal surface. (A) TEM and STEM images of uniform as-deposited ALD coating of CeO_*x*_ and the unimodal structure after electrolysis at 750 °C for 530 h. (B) Chemistry map of different elements of the surface region from the air electrode after 530 h of electrolysis at 750 °C. (C) The schematic of the unimodal structure and nanoionics on SSZ.

To further elucidate the impact of interface constraints on the nanostructure stability of the thin films, contrasting nonsubstrate-constrained and bulk-scale Pr_2_CeO_*x*_ ceramics were synthesized using the sol–gel chemistry route. The grain size of the powders is ∼50 nm after 2 h of calcination at 750 °C, as shown in Figure S1. In contrast, as shown in Figure 2, nanoionics has a grain size of 5–10 nm, even after 530 h of continuous electrolysis at 750 °C. Because the as-deposited ALD film is amorphous, the as-deposited ALD film applied on the substrate that is chemically immiscible with the film is apparently subsequently subjected to further nucleation and crystallization at elevated temperatures. Nevertheless, the 5–10 nm grains within the nanoionics evidently demonstrated that those grains have gone through self-limiting nonequilibrium self-assembly, for which the grain growth appears to have ceased after dwell time at elevated temperatures and formed the nonepitaxial film consisting of nanograins with random crystal orientation.^6,31^ The chemistry immiscibility between the ALD film and the substrate, as well as its related interface strain, presumably played a crucial role in stabilizing the nanograins during electrochemical operation at elevated temperatures.

The formation of conformal surface nanoionics immediately elevated the conductivity of the electrode and the entire cell. For the bimodal structured coating and the nanoionics PrO_*x*_ on SSZ, under electrolysis at 850 °C, the area-specific resistance (ASR) of the cell decreased to 0.1007 Ω cm^2^, representing a 30% reduction, in comparison with that baseline of 0.1469 Ω cm^2^, accompanied by a decrease in both the polarization resistance and ohmic resistance, as shown in Figure 3A. The baseline cell experienced immediate delamination upon SOEC, and the impedance data were obtained only for a zero-hour operation. After electrolysis for 500 h at 850 °C, the ALD-coated cell with a bimodal structure on the oxygen electrode surface exhibits a polarization resistance of 0.0654 Ω cm^2^, which is lower than that of 0.0972 Ω cm^2^ from the baseline cell at 0 h operation, indicating an increase in electrochemical reaction sites on the electrode surface due to ALD coating. The additional ionic conductivity in LSM is warranted due to its alloying with Pr, with a mixed valence state, and the entire surface of (La_0.6_Sr_0.17_Pr_0.22_)­MnO_*x*_ is thus active for the oxygen evolution reaction. Meanwhile, the electrocatalytic conformal nanoionics of PrO_*x*_ on the SSZ effectively transform the YSZ surface into an embedded interface, and the conformal PrO_*x*_ surface nanoionics are electrochemically active, further contributing to extended electrochemical reaction sites and a reduced polarization resistance compared with the baseline.

**Figure 3:**
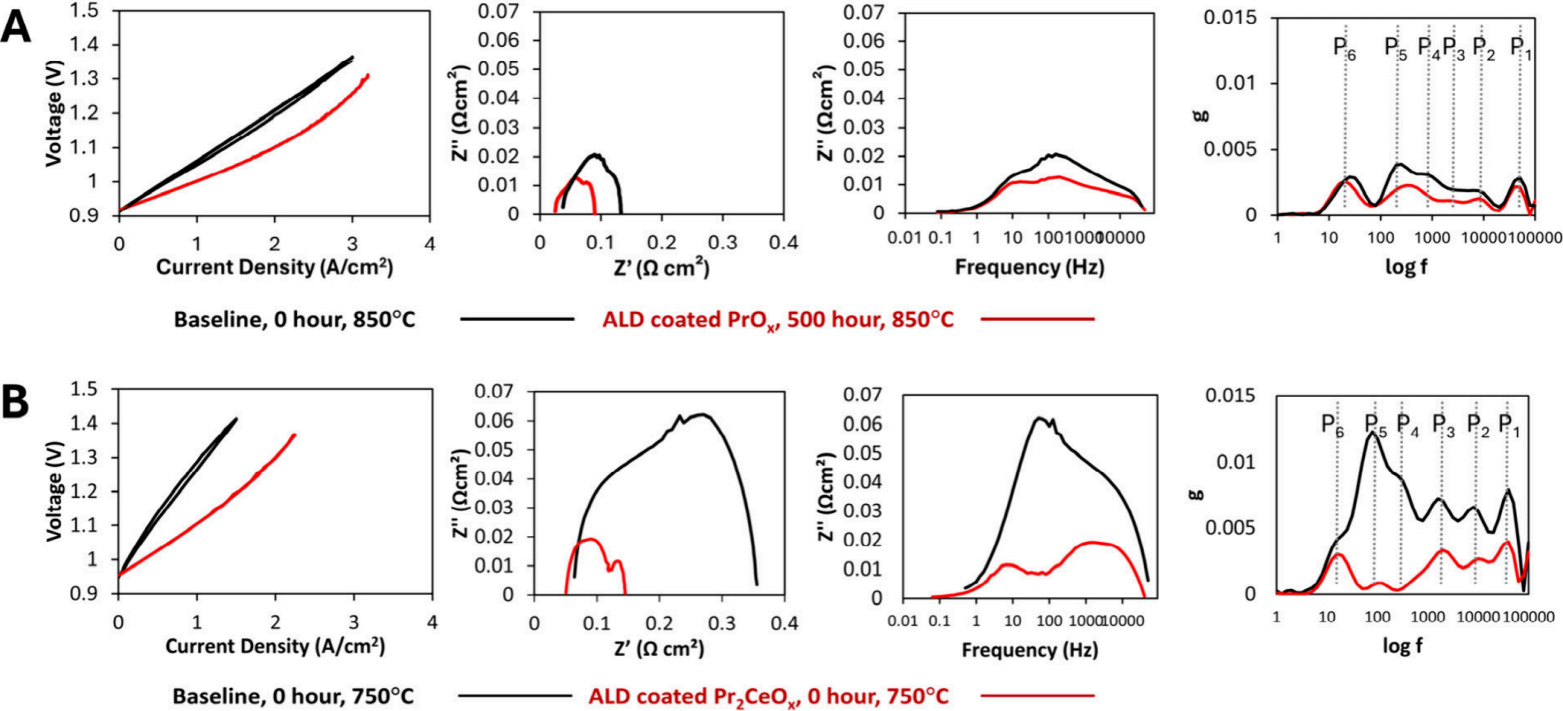
V–I curve, Nyquist plot, Bode plot, and deconvolution spectra of impedance data of the SOEC. (A) Baseline and ALD-PrO_*x*_ coated cells were operated at 850 °C, with the related TEM images shown in Figure 1. (B) Baseline and ALD- Pr_2_CeO_*x*_ coated cells were operated at 750 °C, with the related TEM images shown in Figure 2.

To identify the physical origin of polarization resistance changes, the dynamic constant was retrieved in the impedance data by evaluating the relaxation times and relaxation amplitude using deconvolution.^32–35^ The deconvolution spectrum of cells exhibits six major arcs, with the dominant arcs of P_4_ and P_5_ having frequencies ranging from 100 to 200 Hz and from 20 to 40 Hz, respectively. The peaks of the ALD-coated cell shifted compared to those of the baseline, which is consistent with the change in the LSM chemistry induced by doping Pr from the ALD coating.^36^ Since the baseline and ALD-coated cells possess identical structures from the fuel electrode and electrolyte and operate under the exact conditions, the decreased amplitude of arcs, especially P_4_ and P_5_ in the cell with nanoionics on the air electrode at 100–200 Hz that is primarily associated with oxygen-evolution reactions at the oxygen electrode, is attributable to enhanced oxygen surface exchange and O^2^-ion mass transport within the air electrode and resultant fast electrochemical reactions of the entire cell induced by implantation of surface nanoionics.

Impedance peaks at P_1_, P_2_, and P_3_ above 2500 Hz are associated with overlapping contributions from the fuel electrode, the air electrode, and the electrolyte. The reduction in intensity for peaks P_1_, P_2_, and P_3_ is the direct result of the accelerated mass-transport and fast reaction kinetics induced by the surface engineering modification only on the air electrode with the introduction of a unimodal or bimodal ALD coating.

The ALD-coated cell also has a lower ohmic resistance of 0.0247 Ω cm^2^ compared to that of the baseline cell of 0.0359 Ω cm^2^, shown in Figure 3A. This reduced ohmic resistance and accompanying increased conductivity induced by the ALD coating of electrocatalytic PrO_*x*_ is most striking, and the change of electrical conductivity in LSM induced by Pr alloying is also negligible, as shown in Figure S2. The intragranular conductivity reflected from that of the bulk-scale PrO_*x*_ is very low, and its comparison with LSM and SSZ in conductivity is as follows. (1) Electrical Conductivity: LSM (100–150 S/cm) ≫ PrO_*x*_ (10^–3^ to 10^–2^ S/cm) ≫ SSZ (∼10^–6^ S/cm). (2) Ionic Conductivity: SSZ (0.15–0.18 S/cm) ≫ PrO_*x*_ (∼10^–4^ S/cm) ≫ LSM (∼10^–3^ S/cm). The intergranular defects, including the grain boundaries within the conformal PrO_*x*_ surface nanoionics on the SSZ and the associated interface, are thus considered to be the only source contributing to the increased conductivity of the entire cell. Based on the reduction in ohmic resistance, R_s_, the conductivity of the nanoionic layer on SSZ is calculated to be 1.05 × 10^5^ S/m at 850 °C, which is approximately over 2 orders of magnitude greater than that of LSM in terms of the electrical conductivity and 5 orders of magnitude greater than that of SSZ in terms of the ionic conductivity, as well as 7 orders of magnitude greater than that of bulk scale PrO_*x*_ reported in the literature, as shown in the Supporting Information.

For the cell with unimodal nanoionics, depicted in Figure 2, at 0 h of operation, the ASR value of 0.1796 Ω cm^2^ is obtained for the cell with ALD coating of Pr_2_CeO_*x*_ on the air electrode, compared to 0.3063 Ω cm^2^ for the uncoated cell, representing ∼40% reduction in ASR, as shown in Figure 3B. The reduction in ASR is accompanied by a decrease in both ohmic and polarization resistance. The polarization resistance reduction is dominant, as it decreased from 0.2961 to 0.0946 Ω cm^2^, representing a 68% reduction compared to the baseline. Remarkably, the peak positions of the deconvolution spectra of the impedance of the ALD-coated cell remain the same as that of the baseline, revealing the intact backbone structure, for which TEM shows no interdiffusion between the substrate backbone and the ALD coating layer. The ALD coating transforms the LSM/SSZ internal surface of the entire air electrode backbone into an embedded interface. Consequently, the electrochemical reactions for the ALD-coated cells subsequently occur on the electrocatalytic Pr_2_CeO_*x*_ surface nanoionics, which are conformal on both LSM and SSZ. Such a fully activated internal surface results in a drastic reduction in the peaks at P_4_/P_5_, associated with the O_2_ surface exchange and the O^2–^-ion mass transport within the air electrode for the LSM-coated cell.

The ohmic resistance of the entire cell decreased from 0.0606 to 0.0508 Ω cm^2^, representing a 16% reduction in ALD-coated cells compared to the baseline. Because the electrode backbone remains intact, the reduced ohmic resistance and the induced conductivity are solely due to the unimodal nanoionics. Based on the magnitude of the increased conductivity of the entire cell induced by nanoionics implanted on the surface of the oxygen electrode backbone, the resultant resistivity value for the film is estimated as ρ_nanoionics_ = 1.96 × 10^–3^ Ω cm at 750 °C. The conductivity value for the film is derived to be 5.09 × 10^4^ S/m at 750 °C, as detailed in the Supporting Information. By contrast, the electrical components of the conductivity of bulk-scale Pr_2_CeO_*x*_, with a grain size of ∼50 nm, shown in Figure S1, are only measured as 21.2 S/m at 750 °C in the reducing atmosphere of He, as shown in Figure S3. At 750 °C in air, the comparative conductivity data are as follows. (i) Electrical Conductivity: LSM (50–100 S/cm) ≫ Pr_2_CeO_*x*_ (10^–3^ to 10^–2^ S/cm) ≫ SSZ (∼10^–6^ S/cm). (ii) Ionic Conductivity: SSZ (0.08–0.12 S/cm) ≫ Pr_2_CeO_*x*_ (∼10^–3^ S/cm) ≫ LSM (∼10^–3^ S/cm). The conductivity of the surface nanoionics is thus 2 orders of magnitude higher than that of LSM, 5 orders of magnitude higher than that of the ionic conductivity of SSZ, and 7 orders of magnitude higher than that of bulk-scale Pr_2_CeO_*x*_. Obviously, the grain boundaries within the Pr_2_CeO_*x*_ nanoionics and their interface with the LSM and SSZ substrates exhibit high conductivity, contributing to a decrease in ohmic resistance of the electrode and the entire cell.

The reduction of resistance also simultaneously increases the peak power density of the SOFC mode to 186% and 240% compared with that of the baseline at the temperatures of 850 and 750 °C, respectively, as shown in Figure 4A. This is the largest increase in the SOFC power density induced by electrode surface modification without involving precious metals. The peak power densities of 2.29 W/cm^2^ at 850 °C and 1.81 W/cm^2^ at 750 °C for the cell with an ALD coating on the air electrode are also the highest power densities for commercial cells incorporating ordinary LSM and LSCF electrodes.^37,38^

**Figure 4:**
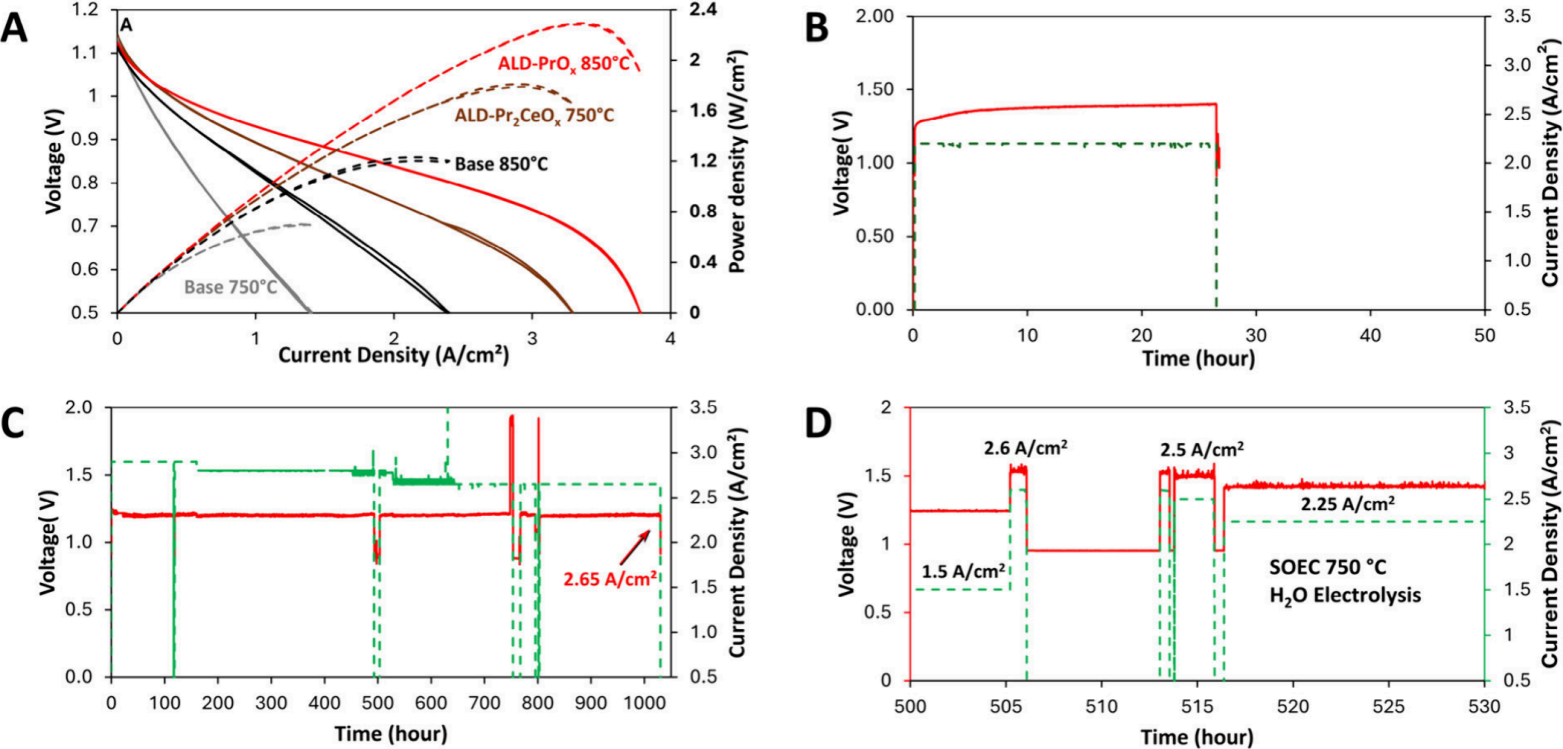
Increase of the SOFC power density and the evolution of the SOEC terminal voltage. (A) Baseline and ALD-coated cells operated under SOFC at 750 and 850 °C. (B) The baseline cell delaminates immediately after SOEC at 2.2 A/cm^2^ for 26 h. (C) ALD-PrO_*x*_-coated cell with bimodal structure, as shown in Figure 1, which exhibited an electrolysis current density of 2.6 A/cm^2^ at 1.2 V during electrolysis at 850 °C for 1000 h. (D) The ALD-Pr_2_CeO_*x*_-coated cell with unimodal nanoionics, as shown in Figure 2, which exhibited an electrolysis current density of 1.5 A/cm^2^ at 1.2 V during electrolysis at 750 °C for 1000 h.

The surface nanoionics have an immediate impact on the long-term electrochemical performance. During electrolysis under a galvanic state with a fixed current density of 2.2 A/cm^2^ at 850 °C, the LSM baseline cell rapidly increases the voltage to approximately 1.4 V after 26 h, as shown in Figure 4C. The electrode delaminates immediately thereafter. Cells with an ALD coating of PrO_*x*_ that exhibit bimodal structures on the air electrode show stable operation at 2.9 A/cm^2^ at 1.2 V at 0 h of operation. After 1030 h of operation at 850 °C, under the same voltage of ∼1.2 V, the cell maintained the high current density of 2.65 A/cm^2^, as shown in Figure 4C. To our best knowledge, for cells reported being tested over 1000 h under continuous electrolysis, this ALD-coated cell possesses, by far, the highest H_2_O electrolysis current density for H_2_ production.^39^ The H_2_ production rate and the related electrolysis current density of cells and stacks reported during the past 15 years are shown in Table S2.

For the cell with unimodal nanoionics, after the operation for 500 h at a voltage of <1.2 V and current density of 1.5 A/cm^2^, the ALD-coated cells were subjected to the dynamic operation at a higher current density of 2.5 A/cm^2^, at the voltage of 1.5 V and then continuous operation at a high current density of 2.25A at 1.4 V, as depicted in Figure 4D. Such dynamic operation and increased electrolysis current density are associated with the variation of the oxygen partial pressure and the accelerated mass and charge transfer that represents the additional thermodynamic driving force for various reactions at elevated temperatures, including grain growth. Nevertheless, after electrochemical operation at 750 °C for 530 h, the randomly oriented 5–10 nm dual-layer nanograins within the uniform and conformal surface nanoionics, as depicted in Figure 2, manifested their complete tolerance toward the high overpotential associated with such dynamic operation. To the best of our knowledge, for cells reported to have been tested over 500 h under continuous electrolysis at 750 °C, this ALD-coated cell possesses, by far, the highest H_2_O electrolysis current density for H_2_ production, as shown in Table S2.

In addition to dramatically boosting the energy conversion performance in both fuel cell and electrolysis modes, the surface nanoionics implanted on the internal surface of the porous air electrode provide a critical insight for practical approaches to enable electrolysis. In particular, for SOFCs that employ the LSM electronic conductor, once subjected to SOEC operation, they commonly inevitably encounter delamination under high electrolysis current density >1 A/cm^2^, and the delamination takes place at the internal interface between the air electrode and electrolyte due to the lack of ionic conductivity of the LSM and presumably the buildup of the local oxygen partial pressure. The present study, especially the unimodal nanoionics simultaneously placed on both the LSM and SSZ, clearly demonstrates that, without altering the physical structure and chemistry of the electrode and without changing the internal interface between the air electrode and electrolyte, the electrode delamination can be immediately mitigated by solely introducing ionic pathways on the internal surfaces of LSM/SSZ. The surface nanoionics thus alter the mobility and conducting pathways of ions, reducing the accumulation of partial oxygen pressure at the internal interface between the electrode and electrolyte. It provides one ultimate solution for mitigating the long-lasting issues of electrode delamination resulting from the lack of an ionic pathway in the LSM.

On the other hand, the air electrode, such as that made of a state-of-the-art LSM composite electrode, is commonly perceived to contribute only 5–10% of the total resistance of the entire cell. The present study demonstrated that the minute amount of ALD coating of PrO_*x*_ and Pr_2_CeO_*x*_ on the internal surface of the as-fabricated air electrode of inherently functional SOFC dramatically decreases the ASR of the whole cell by 30–40% during electrolysis, as shown in Figure 3. The mass-transfer could be further accelerated, and the ASR could be further reduced with the implementation of nanoionics, which consist of well-developed electrocatalysts, such as LSM, that have a higher intragranular conductivity than PrO_*x*_ and Pr_2_CeO_*x*_ at the bulk scale. Unimodal surface nanoionics in Figure 2 depict the stable dual-layered nanoionics that inspire the design of dual-layer nanoionics. Because LSM is immiscible with SSZ, based on the present results, the superjacent LSM nanoionics, with their substantial ionic conductivity from the grain boundaries of LSM, are readily established on the subjacent SSZ nanoionics to form a dual-layered nanoionics, further enhancing cell performance.

The schematic in Figure 5 depicts the potential of dual layers of surface nanoionics consisting of LSM nanoionics that are currently being developed. A 10 nm thick superjacent LSM nanoionics, with conductivity 3 orders of magnitude higher than that of bulk-scale LSM, is grown on the 10 nm thick SSZ nanoionics that are further implanted on the internal surface of the conventional LSM/SSZ backbone. As the grain boundaries within the electrocatalytic LSM nanoionics layer exhibit ionic conductivity,^40–42^ the region for the electrochemical reaction is not only conformal on the LSM/SSZ active layer but also extends to the current-collecting layer. In that case, the ionic pathway will be provided by four different sources of SSZ nanoionics, LSM nanoionics, the interface between the SSZ and the backbone, and the interface between the LSM and SSZ nanoionic layers.

**Figure 5:**
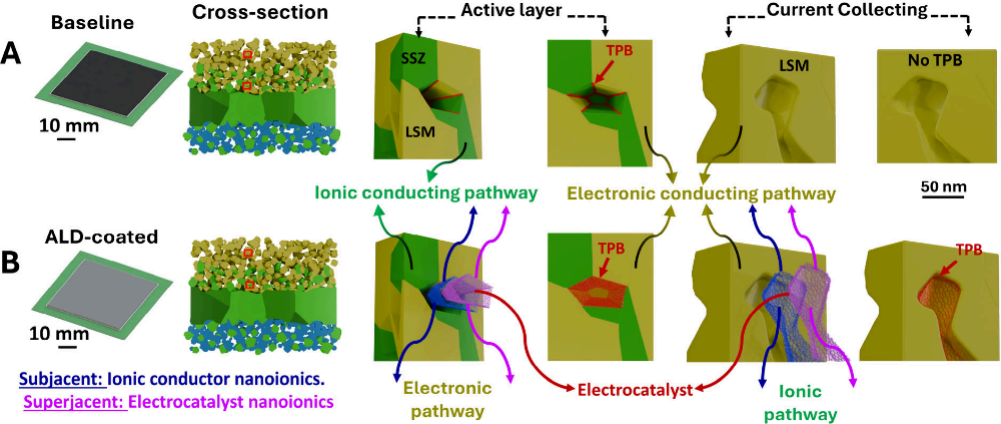
Internal surface structure and TPB regions from both the active layer and the current-collecting layers of the oxygen electrode with layered nanoionics. (A) Baseline. (B) Cell with ALD coating consisting of dual layers of nanoionics, including subjacent SSZ and superjacent LSM, on the air electrode of the cell.

As projected in Figure 6, by further accelerating the mass-transport and applying the unimodal nanoionics to the oxygen electrode alone, the ASR of the entire cell is extrapolated to be less than 0.05 Ω cm^2^, at ∼800 °C, and could lead to the electrolysis at 1.2 V with the hydrogen production rate of ∼5.4 A/cm^2^, which is an order of magnitude higher than what could be provided by the current state of the art SOEC. The surface nanoionics facilitated by ALD coating thus exert a formidable approach for fast oxygen diffusion and swift exchange kinetics, enabled by high-density surface grain boundaries and heterogeneous interfaces.

**Figure 6:**
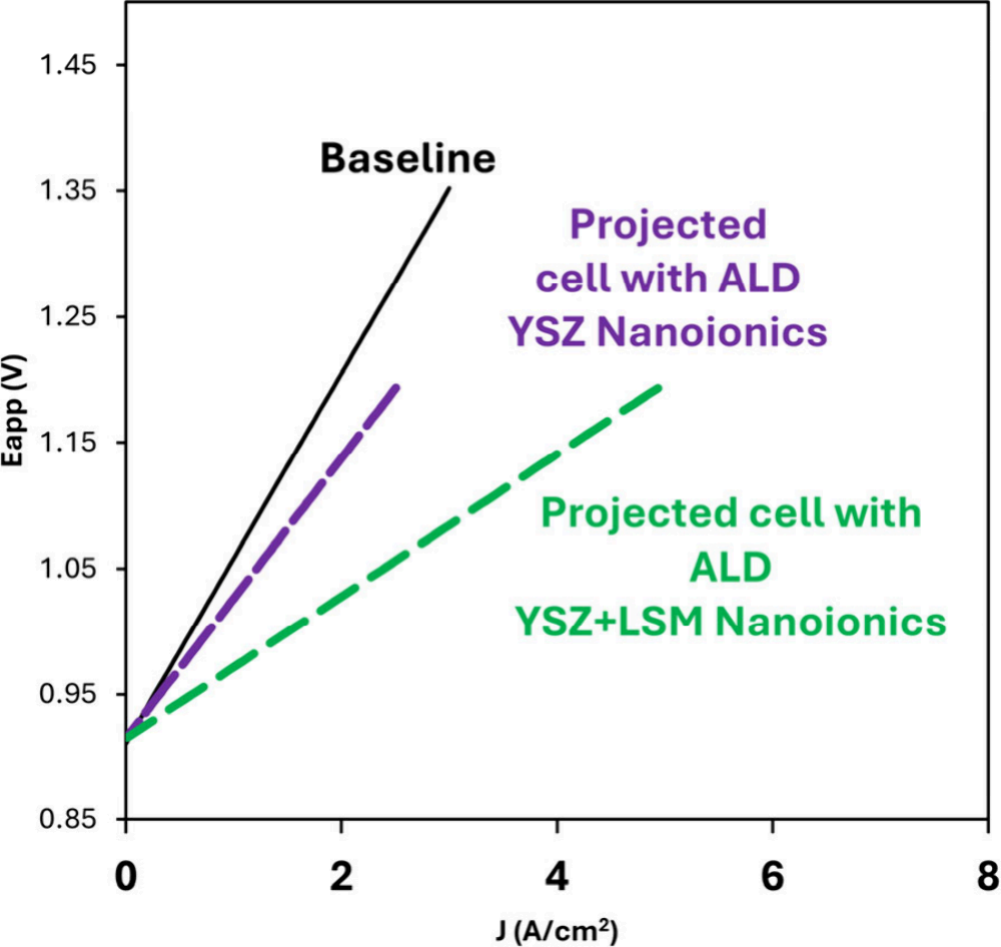
Projected V–I curve under electrolysis at 800 °C for the cell with an ALD coating of dual-layer nanoionics, consisting of subjacent YSZ ionic conductors and superjacent LSM nanoionics, as illustrated in Figure 5.

## Conclusion

In summary, our group has collectively established various surface nanoionics, including ZrO_2_, CoO_*x*_, MnCoO_*x*_, PrO_*x*_, and Pr_2_CeO_*x*_, which have all been proven to be stable during extended electrochemical operations at elevated temperatures. For the first time in the field of electrochemical devices with applications at high temperatures of 750–850 °C, the present study presents a compelling practical approach to prevent the amorphous as-deposited ALD film from forming a solid solution with the substrate, thereby forming conformal thin-film surface nanoionics. It further elucidates the design principle for establishing stable nonepitaxial thin-film nanoionics from various oxides on the substrate by controlling the interfacial immiscibility between the substrate and the film. The nonequilibrium thin-film surface modification facilitated by interface strain, primarily driven by the thinness of the ALD film and substrate–film immiscibility, as identified in the present study, provides a novel approach to exploit the juxtaposition of immiscible phases. The nanoionic film and the paired substrate could be versatile and flexible from the transition-metal oxide to the rare-earth oxide, principally carbide and nitride, which can all be enabled by ALD coating to intentionally create metastable nanoionics-by-design that would be inaccessible through bulk synthesis methods and could revolutionize a wide range of devices, including sensors and reactors for which surface reactivity and conductivity are essential.

## Materials and Methods

Commercially available, anode-supported solid oxide button cells fabricated by Chemtronergy LLC (CTG, Salt Lake City, UT) were employed for all of the experiments described in this paper. CTG cells are composed of five layers as follows, starting from the anode: ∼0.9 mm thick Ni/YSZ cermet layer, which supports the cell structure; 15 μm thick Ni/YSZ active layer; ∼12 μm thick YSZ electrolyte; ∼15 μm thick La_0.8_Sr_0.2_MnO_3_/8YSZ active layer; and 50 μm thick, pure LSM current collecting layer. The active area (limited by the cathode) of the cell is 2 cm^2^. The exposure area of the anode to the fuel is about 3.5 cm^2^.

The ALD coatings were performed in a commercial GEMStar-8 ALD reactor from Arradiance Inc. The precursors used in this study were all purchased from Strem Chemicals, Inc. The tris­(*i*-propylcyclopentadienyl)­cerium­(III) (99.9%) and the ozone were used as the Ce precursor and oxidant for depositing the CeO_*x*_ layer, and the tris­(*i*-propylcyclopentadienyl)praseodymium (99.9%-Pr) and ozone were used as the Pr precursor and oxidant, respectively, for PrO_*x*_ layer growth. During the deposition, tris­(*i*-propylcyclopentadienyl)cerium and tris­(*i*-propylcyclopentadienyl) praseodymium containers were maintained at 165 °C, and the reactor chamber was set at 300 °C. The growth rates are typically ∼0.3 Å/cycle for CeO_*x*_ and ∼0.2 Å/cycle for PrO_*x*_. This is a simple one-step processing of as-received cells, and the change of the chemistry in the ALD layer was achieved through computer-controlled automatic switching of the precursors. No surface pretreatment was applied to the cells, and no heat treatment was applied before or after ALD coating either. The cell electrochemical operation was carried out directly after the ALD coating.

All cell tests were performed on a test stand. The platinum mesh was used for the anode and cathode lead connections. The fuel and air streamflow rates were controlled separately using mass flow controllers. Cell testing was performed at 750 °C. During the operation, an air flow rate of 600 mL/min and a fuel flow rate of 600 mL/min were used. Before any electrochemical measurements, all cells were current-treated for approximately 16 h under a small current density of 0.1 A/cm^2^ to ensure they were activated. After that, all samples were loaded at a constant current of 0.3 A/cm^2^ for the desired periods. The cell performance was examined using a TrueData-load modular electronic DC load, which guarantees voltage and current accuracies of 0.03% FS of the range selected ±0.05% of the value. The cell impedance spectra were examined by using a potentiostat/galvanostat (Solartron 1287A) equipped with a frequency response analyzer (Solartron 1260). Impedance measurements were performed using a Solatron 1260 frequency response analyzer over a frequency range of 50 mHz to 100 kHz. The impedance spectra and resistance (R_s_ and R_p_) presented were measured under a DC bias current of 0.3 A/cm^2^. On a Nyquist plot, R_s_ is determined by the intercept at the higher frequency end and R_p_ is determined by the distance between the two intercepts.

ALD-coated cells were sectioned and subjected to nanostructural and crystallographic examinations using high-resolution (HR) transmission electron microscopy (TEM) and scanning TEM (STEM). All of the TEM examinations were conducted in the cathode active layer. TEM samples were prepared by mechanical polishing and ion milling in a liquid-nitrogen-cooled holder. Electron diffraction, diffraction contrast, and HRTEM imaging were performed using a JEM-2100 instrument operated at 200 kV. The STEM imaging was performed using a Thermo-Fisher Spectra 300 Probe-Corrected S/TEM, operated at 300 kV. Chemical analysis was carried out under TEM using energy-dispersive X-ray spectroscopy (EDS).

## Supplementary Material



## Notes

Additional data and details regarding the nanograins powders after calcination (Figure S1), electrical conductivity of the LSM and Pr-doped LSM at various temperatures (Figure S2), conductivity of the Pr_2_Ce_1_O_*x*_ bulk ceramics at various temperatures (Figure S2), and calculation of the film conductivity (PDF)
