# Supplementary material for: Nanoionics Drastically Accelerating Mass Transfer at Elevated Temperatures over 750 °C
Source: ACS Nano. 2026 Feb 12;20(7):5738–45. doi: 10.1021/acsnano.5c17239 (PMC12947732; doi:10.1021/acsnano.5c17239)
Supplement: Supplementary file 1 [file nn5c17239_si_001.pdf]

supporting information for

Nanoionics Drastically Accelerating Mass Transfer at Elevated Temperatures over 750 °C

Yun Chen, Cesar-Octavio Romo-De-La-Cruz, Fuming Jiang, Sergio Andres Paredes Navia, Xueyan Song*

Department of Mechanical, Materials, and Aerospace Engineering, West Virginia University, Morgantown, WV, 26506, USA

*Corresponding author. Email: xueyan.song@mail.wvu.edu

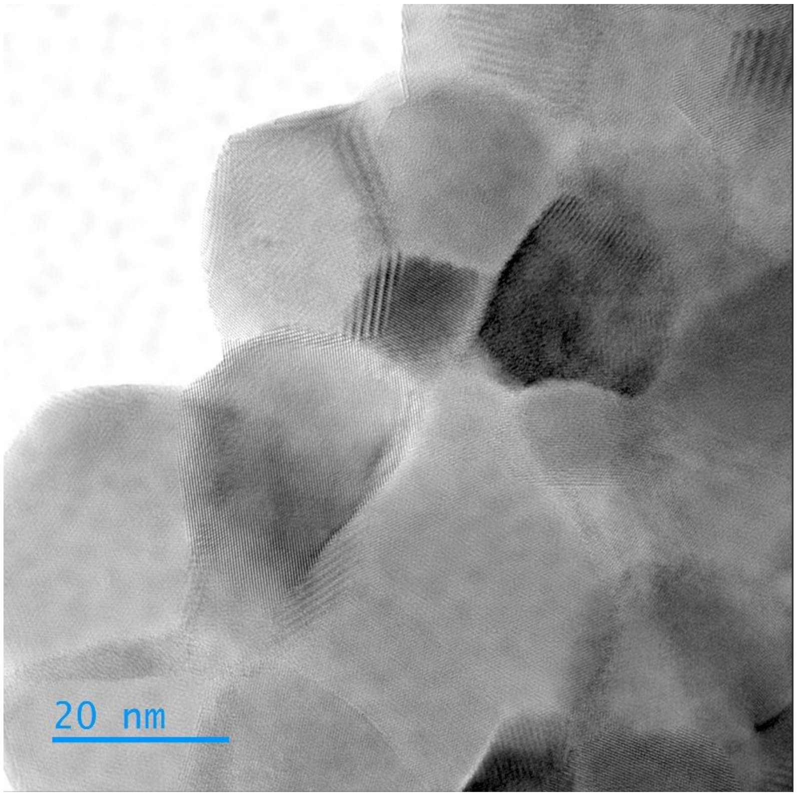

Fig. S1. Nanograins with the size of 20-50 nm upon the calcination at 750°C for only 2 hours.

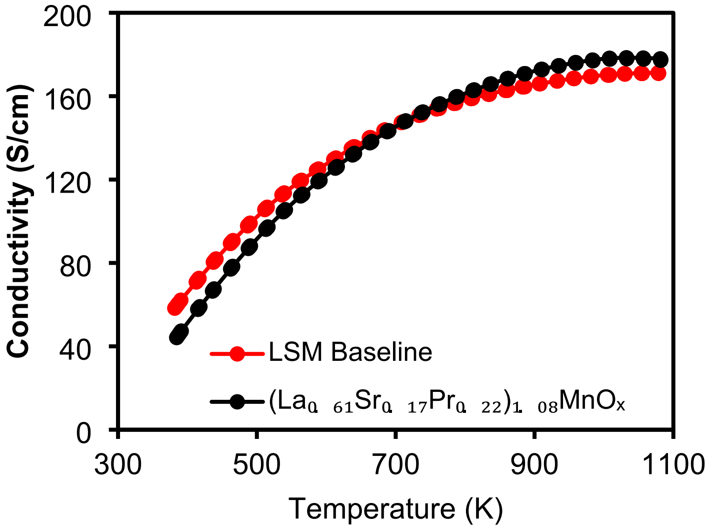

Fig. S2. The electrical conductivity of the LSM and Pr-doped LSM at various temperatures.

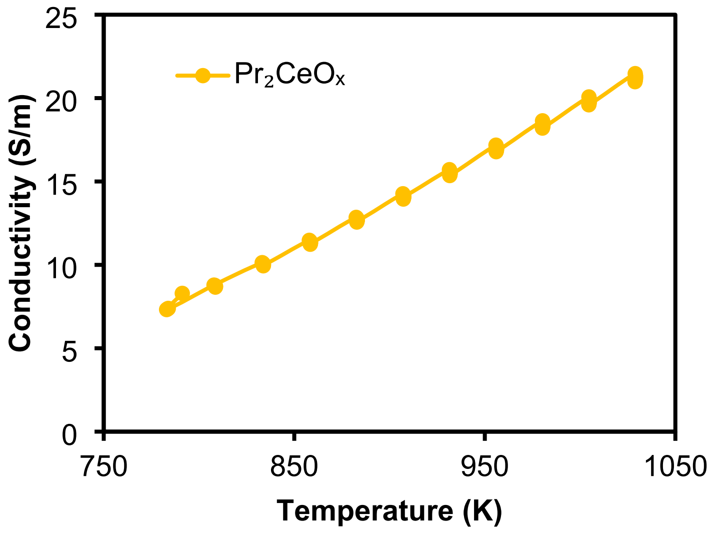

Fig. S3. The conductivity of the $Pr_2Ce_1O_x$ bulk ceramics at various temperatures.

1. Calculation of the conductivity of nanoionics film

In order to get the conductivity of the film, we could use a circuit to describe the contribution of each part of cell (fuel electrode/electrolyte/air electrode).

$$R_{Baseline} = R_{LSM} + R_{LSM/YSZ} + R_{fuel-electrode} + R_{electrolyte} \quad \textbf{Eq. (1)}$$

Where R can be expressed as $Rs\frac{L}{W}$ for each component as:

$$Rs_{Totbase} * \frac{L}{W} = Rs_{LSM} * \frac{L}{W} + Rs_{LSM/YSZ} * \frac{L}{W} + Rs_{fuel-electrode} * \frac{L}{W} + Rs_{electrolyte} * \frac{L}{W} \quad \textbf{Eq. (2)}$$

L/W ratios are in the same magnitude for each component. Thus:

$$Rs_{Totbase} = Rs_{LSM/YSZ} + Rs_{LSM} + Rs_{fuel-electrode} + Rs_{electrolyte} \quad \textbf{Eq. (3)}$$

Eq. (3) corresponding to the uncoated cell has all its components in series. On the other hand, for Eq. (4) we consider the composite of ALD coating and LSM as an equivalent circuit in parallel since the ALD coating is deposited in the LSM cathode. Thus, the equation for the ALD coated cell is as follow:

$$R_{ALDcoated} = R_{composite} + R_{fuel-electrode} + R_{electrolyte} \quad \textbf{Eq. (4)}$$

Where the equivalent circuit for the ALD/LSM contribution $R_{composite}$ is:

$$R_{composite} = R_{LSM} + \frac{1}{\left[\frac{1}{R_{LSM/YSZ}} + \frac{1}{R_{film}}\right]} = R_{LSM} + \frac{1}{\left[\frac{R_{LSM/YSZ} + R_{film}}{R_{LSM/YSZ} R_{film}}\right]} = R_{LSM} + \left[\frac{R_{LSM/YSZ} R_{film}}{R_{LSM/YSZ} + R_{film}}\right] = \frac{L}{W} * R_{LSM} + \frac{L}{W} * \left[\frac{Rs_{LSM/YSZ}\, Rs_{film}}{Rs_{LSM/YSZ} + Rs_{film}}\right] \quad \textbf{Eq. (5)}$$

If we substitute Eq. (5) into Eq. (6):

$$Rs_{ALDcoated} * \frac{L}{W} = R_{LSM} * \frac{L}{W} + \left[\frac{Rs_{LSM/YSZ}\, Rs_{film}}{Rs_{LSM/YSZ}+Rs_{film}}\right] * \frac{L}{W} + R_{fuel-electrode} * \frac{L}{W} + R_{electrolyte} * \frac{L}{W} \quad \textbf{Eq. (6)}$$

L/W ratios are in the same magnitude for each component. Additionally, from the results obtained in the main manuscript we can consider:

$$Rs_{ALDcoated} = Rs_{ratio} Rs_{Totbase} \quad \textbf{Eq. (7)}$$

Where the ratio is defined as the dimensionless ratio between ${Rs_{ALDcoated}}/{Rs_{Totbase}}$.

With the incorporation of Eq. (7) and R expressed as $Rs\frac{L}{W}$ for each component, we have the following equation for the contribution in the ALD-coated cell:

$$Rs_{ALDcoated} = Rs_{LSM} + \left[\frac{Rs_{LSM/YSZ}\, Rs_{film}}{Rs_{LSM/YSZ}+Rs_{film}}\right] + Rs_{fuel-electrode} + Rs_{electrolyte} = Rs_{ratio} Rs_{Totbase} \quad \textbf{Eq. (8)}$$

The only different contributing factors between $Rs_{ALDcoated}$ and $Rs_{Totbase}$ is the LSM and the ALD film. Additionally, by combining $Rs_{anode} + Rs_{electrolyte}$ in Eq. (3) and Eq. (8), we have the following equation:

$$Rs_{Totbase} - Rs_{LSM/YSZ} - Rs_{LSM} = Rs_{ratio}\, Rs_{Totbase} - \left[\frac{Rs_{LSM/YSZ}\, Rs_{film}}{Rs_{LSM/YSZ}+Rs_{film}}\right] - Rs_{LSM} \quad \textbf{Eq. (9)}$$

Further simplification of the Eq. (9) can lead to:

$$\left[\frac{Rs_{LSM/YSZ}\, Rs_{film}}{Rs_{LSM/YSZ}+Rs_{film}}\right] = Rs_{LSM} - Rs_{LSM} + Rs_{LSM/YSZ} - (1 - Rs_{ratio})\, Rs_{Totbase} \quad \textbf{Eq. (10)}$$

$$\left[\frac{Rs_{LSM/YSZ}\, Rs_{film}}{Rs_{LSM/YSZ}+Rs_{film}}\right] = Rs_{LSM/YSZ} - (1 - Rs_{ratio})\, Rs_{Totbase} \quad \textbf{Eq. (11)}$$

$$\frac{Rs_{film}}{Rs_{LSM/YSZ} + Rs_{film}} = (Rs_{LSM/YSZ} - (1 - Rs_{ratio})\, Rs_{Totbase})/Rs_{LSM/YSZ} \quad \textbf{Eq. (11)}$$

$$\frac{Rs_{LSM\,/YSZ} + Rs_{film}}{Rs_{film}} = Rs_{LSM/YSZ}\,/(Rs_{LSM/YSZ} - (1 - Rs_{ratio})\, Rs_{Totbase}) \quad \textbf{Eq. (12)}$$

$$\frac{Rs_{LSM/YSZ}}{Rs_{film}} = [Rs_{LSM\,/YSZ}/(Rs_{LSM\,/YSZ} - (1 - Rs_{ratio})\, Rs_{Totbase})] - 1 \quad \textbf{Eq. (13)}$$

$$Rs_{film} = Rs_{LSM\,/YSZ}/\{[Rs_{LSM\,/YSZ}/(Rs_{LSM/YSZ} - (1 - Rs_{ratio})\, Rs_{Totbase})] - 1\} \quad \textbf{Eq. (14)}$$

We can express each component as $\rho = Rs*t$. Thus, if we replace $Rs = \rho/t$ in Eq. (14), we have the following equation.

$$\rho_{\text{film}} = t_{\text{film}} (\rho_{\text{LSM}/YSZ}/t_{LSM/YSZ}) /\{[(\rho_{\text{LSM}/YSZ}/t_{LSM/YSZ})/((\rho_{\text{LSM}/YSZ}/t_{LSM/YSZ}) - (1 - Rs_{ratio})\, \rho_{\text{Totbase}}/t_{\text{Totbase}})] - 1\} \quad \textbf{Eq. (15)}$$

t_{film} is the thickness of the ALD coating while from the manufacturer, we could obtain the thickness of the LSM and the whole thickness of the cell. The ρ_{LSM} values are obtained from literature while the ρ_{Totbase} is calculated from $Rs = \rho/t$. *Below is the description of the calculation for the experimental data obtained for the LSM cell coated with Pr operated in SOeC mode at 850°C.*

1.1 Calculation for LSM ALD Pr 850C SOEC.

- *For Pr SOeC at 850C, $Rs_{ALDcoated}$ 0.0247 Ω cm2 and Rs_{Base} 0.0359 Ω cm2*
- *Assuming a 15nm thickness for the film, using this value for the actual thickness of the film in calculations.*
- *Assuming only the active layer 10 microns covered with film*
- *Assuming only 50% active layer 10 microns covered with film*
- *Assumption for active layer is 50/50 LSM YSZ*
- *Considering the equation where LSM/YSZ is active layer*

$$\rho_{\text{film}} = t_{\text{film}}(\rho_{\text{LSM}/YSZ}/t_{\text{LSM}/YSZ})/\{[(\rho_{\text{LSM}/YSZ}/t_{\text{LSM}/YSZ})/((\rho_{\text{LSM}/YSZ}/t_{\text{LSM}/YSZ}) - (1 - Rs_{ratio})\rho_{\text{Totbase}}/t_{\text{Totbase}})] - 1\} \quad \textbf{Eq. (15)}$$

*The resistivity value for the film can be calculated employing the Eq. (15). The resultant resistivity value for the film is ρ_{film} =9.56x10^{-4} Ω cm at 850°C, such value represents a ratio of Ratio $\rho_{LSM/YSZ}/\rho_{film}$ of 47.32 at 850°C. Whereas the **conductivity value for the film is 1.05x10^5 S/m at 850°C**. These results show that the ALD coated layer is up to 47.32 times more conductive than the LSM composite backbone, when considering reported literature values (this value is 22.11 of S/cm) for the electrical conductivity of the LSM composite of LSM50% and YSZ8 50% at 850°C.[1] Additionally, the electrical components of the conductivity of Pr6O11 are only ~95 S/m at 850°C. The conductivity of the surface nanoionics is three orders of magnitude of the conductivity of bulk scale Pr6O11, with a **ratio of σfilm / σPr6O11 of ~1101 times larger.**{Ref. Nanoscale research letters(2010) 5, 735-740}*

2. Calculation of film conductivity

In order to get the conductivity of the film we could use a circuit to describe the contribution of each part of cell (fuel electrode/electrolyte/air electrode). The case for the LSM baseline cell will be described by:

$$R_{Baseline} = R_{LSM} + R_{LSM/YSZ} + R_{fuel-electrode} + R_{electrolyte} \quad \textbf{Eq. (1)}$$

Where R can be expressed as $Rs\frac{L}{W}$ for each component as:

$$Rs_{Totbase} * \frac{L}{W} = Rs_{LSM} * \frac{L}{W} + Rs_{LSM/YSZ} * \frac{L}{W} + Rs_{fuel-electrode} * \frac{L}{W} + Rs_{electrolyte} * \frac{L}{W} \quad \textbf{Eq. (2)}$$

L/W ratios are in the same magnitude for each component. Thus:

$$Rs_{Totbase} = Rs_{LSM/YSZ} + Rs_{LSM} + Rs_{fuel-electrode} + Rs_{electrolyte} \quad \textbf{Eq. (3)}$$

Eq. (3) corresponding to the uncoated cell has all its components in series. In the other hand, for Eq. (4) we consider the composite of ALD coating and LSM as an equivalent circuit in parallel since the ALD coating is deposited in the LSM cathode. Thus, the equation for the ALD coated cell is as follow:

$$R_{ALDcoated} = R_{composite} + R_{fuel-electrode} + R_{electrolyte} \quad \textbf{Eq. (4)}$$

Where the equivalent circuit for the ALD/LSM contribution $R_{composite}$ is:

$$R_{composite} = \frac{1}{\left[\frac{1}{R_{LSM}+R_{LSM/YSZ}}+\frac{1}{R_{film}}\right]} = \frac{1}{\left[\frac{R_{LSM}+R_{LSM/YSZ}+R_{film}}{(R_{LSM}+R_{LSM/YSZ})R_{film}}\right]} = \left[\frac{(R_{LSM}+R_{LSM/YSZ})R_{film}}{R_{LSM}+R_{LSM/YSZ}+R_{film}}\right] = \frac{L}{W} * \left[\frac{(Rs_{LSM}+Rs_{LSM/YSZ})Rs_{film}}{Rs_{LSM}+Rs_{LSM/YSZ}+Rs_{film}}\right]$$ **Eq. (5)**

If we substitute Eq. (5) into Eq. (6):

$$Rs_{ALDcoated} * \frac{L}{W} = \left[\frac{(Rs_{LSM}+Rs_{LSM/YSZ})Rs_{film}}{Rs_{LSM}+Rs_{LSM/YSZ}+Rs_{film}}\right] * \frac{L}{W} + R_{fuel-electrode} * \frac{L}{W} + R_{electrolyte} * \frac{L}{W}$$ **Eq. (6)**

L/W ratios are in the same magnitude for each component. Additionally, from the results obtained in the main manuscript we can consider:

$$Rs_{ALDcoated} = Rs_{ratio}Rs_{Totbase}$$ **Eq. (7)**

Where the ratio is defined as the dimensionless ratio between ${Rs_{ALDcoated}}/{Rs_{Totbase}}$.

With the incorporation of Eq. (7) and R expressed as $Rs\frac{L}{W}$ for each component, we have the following equation for the contribution in the ALD coated cell:

$$Rs_{ALDcoated} = \left[\frac{(Rs_{LSM}+Rs_{LSM/YSZ})Rs_{film}}{Rs_{LSM}+Rs_{LSM/YSZ}+Rs_{film}}\right] + Rs_{fuel-electrode} + Rs_{electrolyte} = Rs_{ratio}Rs_{Totbase}$$ **Eq. (8)**

The only different contributing factors between $Rs_{ALDcoated}$ and $Rs_{Totbase}$ is the LSM and the ALD film. Additionally, by combining $Rs_{anode} + Rs_{electrolyte}$ in Eq. (3) and Eq. (8), we have the following equation:

$$Rs_{Totbase} - Rs_{LSM/YSZ} - Rs_{LSM} = Rs_{ratio}\, Rs_{Totbase} - \left[\frac{(Rs_{LSM}+Rs_{LSM/YSZ})Rs_{film}}{Rs_{LSM}+Rs_{LSM/YSZ}+Rs_{film}}\right]$$ **Eq. (9)**

Further simplification of the Eq. (9) can lead to:

$$\left[\frac{(Rs_{LSM}+Rs_{LSM/YSZ})Rs_{film}}{Rs_{LSM}+Rs_{LSM/YSZ}+Rs_{film}}\right] = Rs_{LSM} + Rs_{LSM/YSZ} - (1 - Rs_{ratio})\, Rs_{Totbase}$$ **Eq. (10)**

$$\frac{Rs_{film}}{Rs_{LSM}+Rs_{LSM/YSZ}+Rs_{film}} = [Rs_{LSM} + Rs_{LSM/YSZ} - (1 - Rs_{ratio})\, Rs_{Totbase}]/(Rs_{LSM} + Rs_{LSM/YSZ})$$ **Eq. (11)**

$$\frac{Rs_{LSM}+Rs_{LSM/YSZ}+Rs_{film}}{Rs_{film}} = (Rs_{LSM} + Rs_{LSM/YSZ})/[Rs_{LSM} + Rs_{LSM/YSZ} - (1 - Rs_{ratio})\, Rs_{Totbase}]$$ **Eq. (12)**

$$\frac{Rs_{LSM}+Rs_{LSM/YSZ}}{Rs_{film}} = (Rs_{LSM} + Rs_{LSM/YSZ})/[Rs_{LSM} + Rs_{LSM/YSZ} - (1 - Rs_{ratio})\, Rs_{Totbase}] - 1$$ **Eq. (13)**

$$\frac{1}{Rs_{film}} = \{(Rs_{LSM} + Rs_{LSM/YSZ})/[Rs_{LSM} + Rs_{LSM/YSZ} - (1 - Rs_{ratio})\, Rs_{Totbase}] - 1\}/(Rs_{LSM} + Rs_{LSM/YSZ})$$ **Eq. (14)**

$$Rs_{film} = (Rs_{LSM} + Rs_{LSM/YSZ})/\{(Rs_{LSM} + Rs_{LSM/YSZ})/[Rs_{LSM} + Rs_{LSM/YSZ} - (1 - Rs_{ratio})\, Rs_{Totbase}] - 1\}$$ **Eq. (15)**

We can express each component as $\rho = Rs * t$. Thus, if we replace $Rs = \rho/t$ in Eq. (14), we have the following equation.

$$\rho_{\mathrm{film}} = t_{\mathrm{film}}(\rho_{\mathrm{LSM}}/t_{LSM} + \rho_{\mathrm{LSM/YZS}}/t_{LSM/YSZ})/\{[(\rho_{\mathrm{LSM}}/t_{LSM} + \rho_{\mathrm{LSM/YZS}}/t_{LSM/YSZ})/((\rho_{\mathrm{LSM}}/t_{LSM} + \rho_{\mathrm{LSM/YZS}}/t_{LSM/YSZ}) - (1 - Rs_{ratio})\rho_{\mathrm{Totbase}}/t_{\mathrm{Totbase}})] - 1\}$$ **Eq. (16)**

t_{film} is the thickness of the ALD coating while from the manufacturer, we could obtain the thickness of the LSM and the whole thickness of the cell. The ρ_{LSM} and $\rho_{\mathrm{LSM/YSZ}}$ values are obtained from literature while the ρ_{Totbase} is calculated from *Rs= ρ/t. Below is the description of the calculation for the experimental data obtained for the LSM cell coated with Ce and Pr operated in SOeC mode at 750° C.*

2.1 Calculation for LSM ALD Ce+Pr 750C SOEC.

- *For LSM ALD Ce+Pr SOeC at 750C,* $Rs_{ALDcoated}$ *0.050 Ω cm2 and* Rs_{Base} *0.0601 Ω cm2*
- *Assuming a 13nm thickness for the film(3nmCe+10nm Pr), using this value for the actual thickness of the film in calculations.*
- *Assuming active layer 10 microns and 40 microns current collector layer covered with film*
- *Assumption for active layer is 50/50 LSM YSZ, assumption for current collector pure LSM*
- *Considering the equation for both LSM current collector and LSM/YSZ is active layer*

$$\rho_{\mathrm{film}} = t_{\mathrm{film}} (\rho_{\mathrm{LSM}}/t_{LSM} + \rho_{\mathrm{LSM/YZS}}/t_{LSM/YSZ})/\{[(\rho_{\mathrm{LSM}}/t_{LSM} + \rho_{\mathrm{LSM/YZS}}/t_{LSM/YSZ})/((\rho_{\mathrm{LSM}}/t_{LSM} + \rho_{\mathrm{LSM/YZS}}/t_{LSM/YSZ}) - (1 - Rs_{ratio})\rho_{\mathrm{Totbase}}/t_{\mathrm{Totbase}})] - 1\} \quad \textbf{Eq. (16)}$$

The resistivity value for the film can be calculated employing the Eq. (16). The resultant resistivity value for the film is ρ_{film} *=1.96x10*$^{-3}$ *Ω cm at 750°C, such value represents a ratio of Ratio* $\rho_{LSM/YSZ}/\rho_{film}$ *of 23.07 at 750°C. Whereas the **conductivity value for the film is 5.09x10*^{4} ***S/m at 750°C**. These results show that the **ALD coated layer is up to 23.07 times more conductive than the LSM composite** backbone, when considering reported literature values (this value is 22.07 of S/cm) for the electrical conductivity of the LSM composite of LSM50% and YSZ8 50% at 750°C.[1] Additionally, the electrical components of the conductivity of Pr2CeOx are only 21.2 S/m at 750°C. The conductivity of the surface nanoionics is three orders of magnitude of the conductivity of bulk scale Pr2Ce1Ox, with a **ratio of σfilm / σPr2Ce1Ox of ~2401 times larger.***

2.2 Calculation for hypothetical 40nm YSZ stabilizer coating on LSM at 850°C SOEC.

Assumptions for 40nm YSZ

- *Assuming a 40nm thickness for the ALD film, using this value for the actual thickness of the film in calculations.*
- *For SOeC at 850C,* Rs_{Base} *0.03591 Ω cm2.*
- *Reference for performance of LSM coated with ALD YSZ SOeC at 850° C, Rp is 0.0688 Ω cm2, Rt is 0.1041Ω cm2, and ASR is 0.1130Ω cm2. Rp is reduced from the baseline value of 0.0972 Ω cm2. Nonetheless, for a 40nm ZrO2 it was reported that the Rp could be decreased 30.6% from the baseline values.[2] Assuming the nanoionic size will keep similar performance with 30.6% reduction of Rp.*
- *Assuming active layer 10 microns and 40 microns current collector layer covered with film to stabilize the subsequent layer.*
- *Assumption for active layer is 50/50 LSM YSZ, assumption for current collector pure LSM*
- *Considering the equation for both LSM current collector and LSM/YSZ is active layer*

$$\rho_{\mathrm{film}} = t_{\mathrm{film}} (\rho_{\mathrm{LSM}}/t_{LSM} + \rho_{\mathrm{LSM/YZS}}/t_{LSM/YSZ})/\{[(\rho_{\mathrm{LSM}}/t_{LSM} + \rho_{\mathrm{LSM/YZS}}/t_{LSM/YSZ})/((\rho_{\mathrm{LSM}}/t_{LSM} + \rho_{\mathrm{LSM/YZS}}/t_{LSM/YSZ}) - (1 - Rs_{ratio})\rho_{\mathrm{Totbase}}/t_{\mathrm{Totbase}})] - 1\} \quad \textbf{Eq. (16)}$$

The resistivity value for the film can be assumed to be 3000 times higher that bulk sample(assumed average improvement for YSZ based films), by considering YSZ with a with conductivity value of ~0.0725 S/cm or 7.25 S/m, we could perform backward calculation to get the resultant resistivity value for the film considering a coating of 10nm throughout the air electrode, which will yield a value of ρ_{film} *=4.59x10*$^{-3}$ *Ω cm at 850°C, such value represents a ratio of Ratio* ρ_{LSM}/ρ_{film} *of 9.84 at 850°C. The assumed conductivity to be 3 orders of magnitude higher than that of the bulk, with the film having a value of 2.18x10*^{4} *S/m at 850°C. These results show that using a conventional YSZ coated layer is up to 9.84 times more conductive than the LSM composite active layer, when considering reported literature values (22.11 S/cm) for the electrical conductivity of the LSM composite of LSM50% and YSZ8 50% at 850°C.*[1] *The resultant Rs decrease with the assumed YSZ conductivity conformal film will lead to a 36.5**% reduction of the Rs** for the ALD coated cell compared to that of Rs for the LSM Baseline, with values of **Rs 0.02277 Ωcm2 obtained for the cell with ALD coating of YSZ compared to the Rs for the LSM baseline having a value of 0.0359 Ωcm2**.*

2.3 Calculation for hypothetical performance of LSM with ALD YSZ+LSM coating at 850°C SOEC.

Assumptions for subsequent 10nm LSM on top of YSZ

- *Assuming a 10nm thickness for the ALD film, using this value for the actual thickness of the film in calculations, even a modest reduction of ~2 orders of magnitude(70 times) the calculated value of Rs for 40nm YSZ films coated on LSM cells.*
- *For SOeC at 850C,* Rs_{Base} *0.03591 Ω cm2.*
- *For SOeC at 850C, Rs of YSZ 10 nm is **0.02277 Ωcm2***
- *Rp was reduced from the baseline value of 0.0972 Ω cm2 to a value of 0.0688 Ω cm2 after 10nm YSZ coating, or a 29.2% reduction. Assuming the nanoionic LSM 10nm coating size can reduce in a similar trend the Rp with a 29.2% reduction.*
- *Assuming active layer 10 microns and 40 microns current collector layer covered with film to stabilize the subsequent layer.*
- *Assumption for active layer is 50/50 LSM YSZ, assumption for current collector pure LSM*
- *Considering the equation for both LSM current collector and LSM/YSZ is active layer*

$$\rho_{\text{film}} = t_{\text{film}}(\rho_{\text{LSM}}/t_{LSM} + \rho_{\text{LSM/YZS}}/t_{LSM/YSZ})/\{[(\rho_{\text{LSM}}/t_{LSM} + \rho_{\text{LSM/YZS}}/t_{LSM/YSZ})/((\rho_{\text{LSM}}/t_{LSM} + \rho_{\text{LSM/YZS}}/t_{LSM/YSZ}) - (1 - Rs_{ratio})\rho_{\text{Totbase}}/t_{\text{Totbase}})] - 1\}$$ **Eq. (16)**

The resistivity value for the film can be assumed to be 70 times higher that bulk sample, by considering pure LSM composite with a with conductivity value of ~180.59 S/cm, we could perform backward calculation to get the resultant resistivity value for the film considering a 10nm LSM coating throughout the air electrode, which will yield a value of ρ_{film} *=6.46x10*$^{-4}$ *Ω cm at 850°C, such value represents a ratio of Ratio* ρ_{LSM}/ρ_{film} *of 70 at 850°C. The assumed conductivity to be two orders of magnitude higher than that of the bulk, with the film having a value of 1.55x10*^{5} *S/m at 850°C. These results show that using a conventional 10nm LSM coated layer is up to 70 times more conductive than the LSM composite active layer, when considering reported literature values (22.11 S/cm) for the electrical conductivity of the LSM composite of LSM50% and YSZ8 50% at 850°C. The resultant Rs decrease with the assumed LSM conformal film will lead to a 99**% reduction of the Rs** for the ALD coated cell compared to that of Rs for the LSM Baseline, with values of **Rs 0.0001 Ωcm2***

obtained for the cell with ALD coating of YSZ compared to the Rs for the LSM baseline having a value of 0.0359 Ωcm2.

2.4 Calculation for hypothetical performance of LSM with ALD YSZ+LSM coating at 850°C SOEC.

*The obtained conductivity calculation is used to provide a prediction of the performance for the LSM with ALD YSZ+LSM coating at 850°C during the SOeC operation. From the experimental results we know the Rs, Rp, Rt, and ASR values for the LSM baseline cell operated at 850°C. Additionally, the obtained data for a set of LSM coated via ALD with YSZ can provide a base scenario for the predicted nanoionic layer performance improvement of 29.2% of Rp. Taking the calculated **Rs values for the standalone 40nm YSZ and the multilayer nanoinics comprised of 40nm YSZ and 10nm LSM**, we can predict the ASR for the SOEC operation at 850C and 50% steam. **Rt** values are used to obtain the ASR value for the cell and plot the IV curves. Below is the predicted performance in comparison to the LSM baseline, the LSM Pr coated via ALD, and the projected nanoionics. Table shows the SOeC performance at 1.2V electrolysis voltage.*

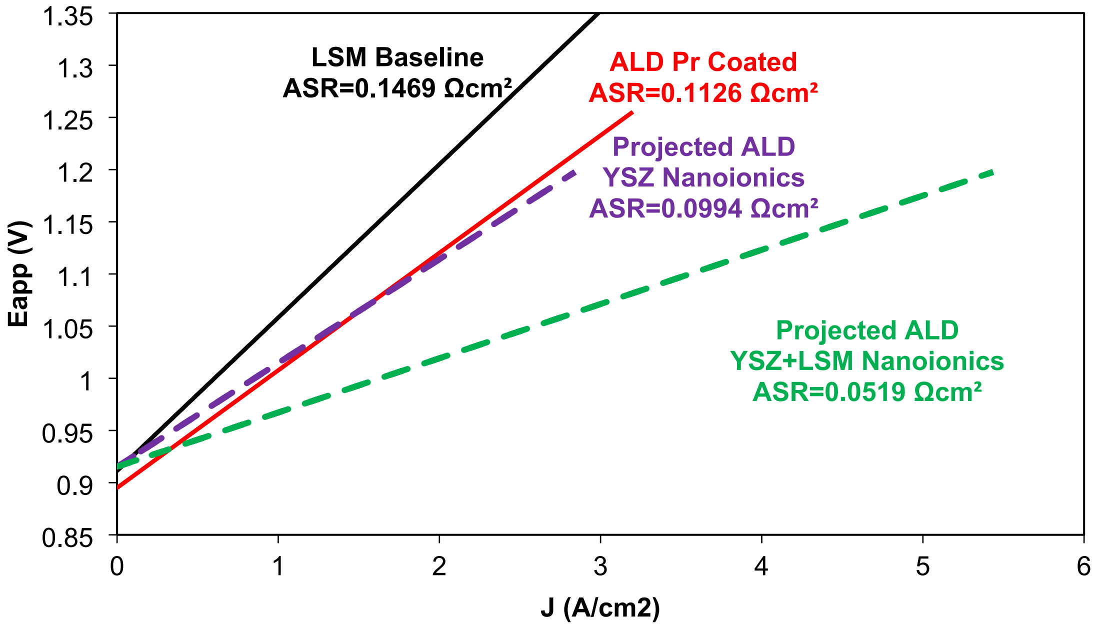

Fig. S4. *The V-I curves under electrolysis at 850°C of the baseline and three of the ALD-coated cell comparing the ALD coating of dual-layer nanoionics consisting of subjacent YSZ ionic conductors with superjacent LSM nanoionics.*

Table S1. Numerical values obtained from *V-I curves under electrolysis at 850°C for the baseline and three of the ALD-coated cell.*

Operation time	Electrolysis Voltage (V)	Current density (A/cm²)
850C SOeC 0h LSM Base	1.2	2.05
850C SOeC 0h LSM ALD Pr	1.2	2.71
850C SOeC LSM ALD 40nm YSZ nanoionics	1.2	2.84

850C SOeC LSM ALD 40nmYSZ+10nmLSM nanoionics	1.2	5.43

Table S2. State-of-the-art performance for SOeC operation in comparable systems

Ref. Year	Cell Material	Cell Type	Operation Mode	Operation temperature (°C)	Run-time (h)	Long-term Opearting Current density (A/cm2)	Current density (A/cm2)	Voltage (V)	Fuel Gas content	Degradation rate
[3]2025	LSCF-CGO/3YSZ/CGO/Ni-CGO	Single cell	H2 CO2 co-electrolysis	860	1000	-0.92			steam/carbon ratio=2 63.7% h2o	4.9 %/kh Vm
					450-1550	−0.25				6.4Vm (0.67 %/kh)
					480-1630	−0.5				5.2Vm (0.47 %/kh)
					480-1630	−1				13 Vm (0.97 %/kh)
[4]2025	LSM-YSZ-20 wt%Ce0.95Cu0.05O2-δ/YSZ/Ni-YSZ	Single cell	SOEC	800			2.6	1.3	50%	N/A
					120	0.2		1.1		
							1	1.1		
[5]2024	LSCF-GDC/GDC Thin/YSZ/GDC Thin/Ni-GDC	Stack 30 repeat units	SOFC	N/A	5200	N/A	N/A		N/A	N/A
			SOEC	846	5200-12000	-0.41	-0.41		80%	0.9%/kh Vm
[6]2023	LSCF/La0.8Sr0.2Ga0.8Mg0.2O3-δ (LSGM)/GDC/ Sr1.95Fe1.4Co0.1Mo0.4Ti0.1O6-δ Co@SFCMT	Single cell	CO2 electrolysis	750	200	1		1.5	10%-CO/CO2	N/A
				750	0		~1	1.5		
				800	0		2.57	1.8		
[7]2023	LaNi0.6Fe0.4O3-δ-La0.45Ce0.55O2-δ (LNF-LDC)@LSM-YSZ/YSZ/Ni-YSZ	Single cell	SOEC	800	0	N/A	1.18	1.3	50%	N/A
[8]2023	LSCoF–GDC/LSCGM/ Sr2Fe1·3Cu0·2Mo0·5O6–δ SFCuM	Single cell	CO2 electrolysis	800	0		1.84	1.5	N/A	
				750	200	1.3	1.68	1.5		no obvious change
				750			1.1	1.5		
[9]2023	Pd–La0.6Sr0.4Co0.2Fe0.8O3–δ./GDC/YSZ/Ni-YSZ	Single cell	SOEC	800	192		1.67	1.5	50%	N/A
[10]2023	Ba0.5Sr0.5Co0.8Fe0.2O3-δ-Gd0.2Ce0.8O2-δ (BSCF-GDC)/GDC/YSZ/NiO-YSZ	Single cell	SOEC	800	0		-4.3	1.5	50%	N/A
[11]2023	LSC-GDC/YSZ/Ni-YSZ	Single cell	SOEC	800	0		-1.86	1.3	50%	
					0		-2.3	1.5		
					0		~0.9	1.02		0.03 mV/h

[12]2022	LSC/GDC/YSZ/NiO-YSZ	Single cell	H2 CO2 co-electrolysis	800	1000		-1		40% H2O + 40% CO2	33%/kh Vm
	LSC/GDC/YSZ/NiO-YSZ	Single cell	SOEC	800	1000	0.59	-1		50%	22%/kh Vm
			SOEC	750	1000		-1		50%	16%/kh Vm
[13]2022	PCFC/GDC/YSZ/NiO/YSZ	Single cell	SOEC	850	0		2.23	1.3	50%	N/A
				800			0.9	1.2		
				750			0.79	1.3		
			rSOFC/rSOEC	800	200	0.4 SOEC -0.4 SOFC		1.2	N/A	no obvious change
[14]2021	LCaFN-GDC/YSZ/Ni-YSZ	Single cell	SOEC	850	0		1.39	1.3	70%	N/A
				800			1.2			
				800			~0.5	1.1	N/A	good reversibility and stability.
			rSOFC/rSOEC	800	12	0.4 SOEC -0.4 SOFC		~1.1		
[15]2021	LSM-YSZ/YSZ/Ni-YSZ	Single cell	SOEC	800	0		1.26	1.3	50%	
				800			~0.5	1.2		
				750	120	-0.5		~1.2		
[16]2021	Pr0.6Sr0.4CoO3-δ (PSC)/GDC/ YSZ/Ni-YSZ	MELLEN (Single stack)	SOFC	650	17,820			0.85 V	46%	2.4%/kh A/cm2, 1.1% /kh A/cm2 after 9kh
			SOEC	800	860			1.5 V	46%	16%/kh A/cm2
[17]2021	LSCF-GDC/YSZ/GDC/Ni-YSZ	Tubular cells	SOEC	800	1000	-0.5			80%	16%/kh V
	LSCF+GDC-Graphite 6%/YSZ/GDC/Ni-YSZ		SOEC	800	1000	-0.5		~1.2	80%	7.1%/kh V
	LSCF+GDC-Graphite 6%/YSZ/GDC/Ni-YSZ			800	0		0.7	1.2		
[18]2020	LSCF/GDC/3YSZ/NiO-GDC	Stack	SOFC	856	200	0.5			75%	
			SOEC	856 °C (1 kh) to 860 °C (8.7 kh)	8756 h on time: 5800h switching time: 2700 h	on -0.7, off -0.07		~1.2	75%	0.4%/khr V
					0		0.7	1.1		
[19]2020	LSCF-GDC/YSZ/GDC/NiO-GDC		SOEC	820	3370	-0.52			80%	0.5%/kh voltage

		30-cell stack	rSOFC/rSOEC	820(SOEC) 750(SOFC)	2500	-0.38 to -.52 SOEC 0.18 SOFC			80%	–0.14%/kh SOEC v –1.3%/kh SOFC v
[20] **2020**	LSFMo/LSGM/LSFMo	Symmetric	SOEC	850	0		2	1.6	20%	
			rSOFC/rSOEC	850	90	0.5 SOEC -0.5 SOFC		~1.2	20%	without detectable degradation
					0		0.79	1.2		
[21] **2020**	LS46M-GDC/YSZ/Ni-YSZ	Single cell	SOEC	850	0		2.24	1.3	40%	
			SOEC	800			1.3	1.25		
			SOEC	800	20	1		~1.25	20%	
			rSOFC/rSOEC		31	0.8 SOEC -0.4 SOFC				
[22] **2020**	LSC infiltrated CGO /YSZ/NiO-YSZ	Single cell	SOEC	800	0		1.07	1.3	50%	
				750	86	-0.5		~1.075	90%	~0.3 to ~0.6 mV h−1 in the first 30 h 0.1 mV h−1 in the following 50 h
[23] **2020**	LSF/GDC/YSZ/Ni-YSZ	Single cell	H2 CO2 co-electrolysis	800	0		1.52	1.3	Dry	
				750	0		0.98	1.3		
[24] **2020**	LSCF-GDC/GDC/YSZ/Ni-YSZ	Single cell	SOFC	800	1000	1			50%	0.8%/1000 h V
			SOEC		1000	-1	-1	~1.25		8.3%/1000 h V
[25] **2020**	LSM-YSZ/YSZ/Ni-YSZ	Single cell	CO2 electrolysis	800	0		3.1	1.5	CO2–CO (90–10 vol %)	
					0		6.3	1.8		
					20	1.25		~1.5		
[26] **2019**	LSCF-CGO/YSZ/CGO/Ni-YSZ	Single cell	SOEC	800	4400	-1		~1.3	90%	25 mV/khr (250–4,400 h)
					after 4400		0.9	1.3		
[27] **2019**	PrO-SDC/ScCeSZ/Ni-SDC	Single cell	SOEC	800	0		−5.31	1.3	50%	
				750			−4.09	1.3		
[28] **2019**	Sm0.5Sr0.5CoO3-Ce0.8Sm0.2O1.9/YSZ/NiO-YSZ	Single cell	SOEC	800	0		4.08	1.3	50%	
				750			2.3	1.2		
				750	40	2		~1.2	40%	

[29]**2019**	STFC infiltrated LSM-YSZ /YSZ/Ni-YSZ	Single cell	SOEC	800	0		1.3	1.3	50%	
	STFC infiltrated LSM-YSZ /YSZ/STFC infiltrated LSM-YSZ	Symmetric		750	800	0.5		0.8	50%	8% increse in ASR
	STFC infiltrated LSM-YSZ /YSZ/Ni-YSZ	Single cell	SOFC	700	250	0.5			3%	
[30]**2018**	LSCF/GDC/8YSZ/Ni-YSZ	F10-design stack	SOEC	800°C	2400	-0.5			50%	0.7% voltage
				700°C	2300					1.9% voltage
				800°C	500					0.8% voltage
				750°C	700					1.9% voltage
				800°C	1500					<0.1% voltage
				800°C	10000					0.4% voltage
				800°C	1300					1% voltage
				800°C	1300					<0
[31]**2018**	NdBa0.5Sr0.5Co1.5Fe0.5O5+δ (NBSCF-BZCYYb)/BaZr0.1Ce0.7Y0.1Yb0.1O3-δ (BZCYYb)/NiO-BZCYYb	Single cell	Hybrid-SOEC (electrolysis at both air and hydrogen electrodes)	750	0		3.16	1.3	10%	
				550			0.42	1.3		
				550		0.45		1.25		
[32]**2017**	LSCF-GDC-0.5Sr0.5CoO3-δ/GDC/YSZ/Ni-YSZ	Single cell	SOFC	750	0-200	0.5			3%	no performance degradation in fuel cell mode for 200 h and in electrolysis mode for 300 h
			SOEC	750	200-500	1.8		~1.25	50%	
			SOEC	750	0		2.1	1.29	50%	
							1.8	1.25		
[33]**2016**	PBSCF-GDC/LSGM/PBM (Co-Fe)	Single cell	SOEC	800	50		1.31	1.3	10%	
				700			0.52	1.3		
			SOEC	700	600	-0.25	0.4	~1.25	10%	without observable degradation
[34]**2016**	LSM-SSZ/SSZ/LCNT	Single cell	SOEC	900	0		2.75	1.3	50%	
				800	0		0.95	1.3	50%	
			SOEC	700	120			0.7	3%	Electrochemical activity do not degrade
[35]**2016**	LSCN-GDC/YSZ/Ni-YSZ	Single cell	SOEC	800	0		1.403	1.5	60%	
				750			-0.5	1.1		

				750	100	-0.4		~1.1		
[36]**2015**	LSCF/GDC/8YSZ/Ni-YSZ	F10-design stack	SOEC	800	2300	-0.5			50%	0.7%/kh v
				700	after 2300					1.9%/kh reduced to 1.1%/kh v
[37]**2015**	LSCF/GDC/6Sc1CeSZ/GDC/NiO-GDC	Stack	SOEC	834	1050	-0.5			75%	0.56%/kh voltage
				843	1461	-0.7			75%	
				847-848	>8600	-0.9		~1.2	75%	
[38]**2015**	LSCF/bi-layered-ScSZ/GDC electrolyte/Ni-YSZ	Single cell	SOEC	800	0		2.2	1.5	80%	
[39]**2014**	LSC-GDC/ScSZ/Ni-YSZ	200 W-SOC Stack	SOEC	750	1000	-0.31			30%	7.75% Vm
				750	120	-1.1		~1.3	50%	
[40]**2014**	LSCF-YSZ/YSZ/LSCF Ni-YSZ	Single cell	SOEC	800			1.14	1.3	50%	
				750			0.98	1.3		
				750	16	0.6 SOEC -0.6 SOFC		~1.2		
							0.5	1.2		
[41]**2014**	LSCF-GDC/GDC/YSZ/NiO-YSZ	Single cell	SOEC	750	0		1.80	1.3	50%	
[42]**2013**	La–Co–Fe oxide based/ScSZ/Ni-Ceria	10-cell Ceramatec stacks	SOEC	800	1000	0.25			56%	5.66%/khr ASR
				800	1000	0.317			56%	4.62%/khr ASR
				800	1900	0.25			56%	
	LSCF/YSZ/Ni-YSZ	5-cell MSRI stack		800	1000	0.2		~4.75	70%	3.7%/khr ASR
					0	0.25		4.75		
[43]**2010**	LSM-YSZ/YSZ/Ni-YSZ	Single cell	SOEC	750	0		0.6	1.3	50% at 10 bar pressure	

References

[1] Paydar, S., Shariat, M. H., & Javadpour, S. (2016). Investigation on electrical conductivity of LSM/YSZ8, LSM/Ce0.84Y0.16O0.96 and LSM/Ce0.42Zr0.42Y0.16O0.96 composite cathodes of SOFCs. International Journal of Hydrogen Energy, 41(48), 23145–23155.

[2] Chen, Y., Gerdes, K., & Song, X. (2016). Nanoionics and nanocatalysts: conformal mesoporous surface scaffold for cathode of solid oxide fuel cells. Scientific reports, 6(1), 32997.

[3] Riegraf, Matthias, Alexander Surrey, Noriko Sata, and Rémi Costa. "Cerium silicate formation in solid oxide electrolysis cells: Effects on durability and mitigation strategies." Chemical Engineering Journal (2025): 164654.

[4] Wang, Yutong, Zhe Zhao, Cheng Bi, Shuai Tang, Xinyi Liu, Ting Yang, Mingdeng Wang, Mojie Cheng, and Zhigang Shao. "Boosting the electrocatalytic activity of LSM-YSZ by loading high catalytic Ce1-xCuxO2-δ for solid oxide electrolysis cells." International Journal of Hydrogen Energy 109 (2025): 918-926.

[5] Han, Feng, Michael Lang, Patric Szabo, Christian Geipel, Christian Walter, and Rémi Costa. "Performance and degradation of electrolyte Supported SOECs with advanced Thin-film Gadolinium doped Ceria barrier Layers in Long-term Stack Test." Journal of The Electrochemical Society 171, no. 5 (2024): 054515.

[6] Lu, Chengyi, Chunming Xu, Wang Sun, Rongzheng Ren, Jinshuo Qiao, Zhenhua Wang, Kening Sun, Guang Pan, and Yonghui Cao. "Enhancing catalytic activity of CO2 electrolysis by building efficient and durable heterostructure for solid oxide electrolysis cell cathode." Journal of Power Sources 574 (2023): 233134.

[7] Tang, Shuai, Zhe Zhao, Chao Zhang, Kun Wang, and Zhigang Shao. "High-performance LaNi0. 6Fe0. 4O3-δ-La0. 45Ce0. 55O2-δ nanoparticles co-loaded oxygen electrode for solid oxide steam electrolysis." Ceramics International 49, no. 2 (2023): 1636-1645.

[8] Xu, Chunming, Lihong Zhang, Wang Sun, Rongzheng Ren, Xiao Yang, Xiaoxia Yang, Minjian Ma, Jinshuo Qiao, Zhenhua Wang, and Kening Sun. "Building efficient and durable 3D nanotubes electrode for solid oxide electrolytic cells." Journal of Power Sources 556 (2023): 232479.

[9] Yang, Rui, Yunfeng Tian, Yun Liu, Jian Pu, and Bo Chi. "Pd–La0. 6Sr0. 4Co0. 2Fe0. 8O3–δ composite as active and stable oxygen electrode for reversible solid oxide cells." Journal of Rare Earths 41, no. 4 (2023): 599-604.

[10] Zhao, Zhe, Shuai Tang, Xinyi Liu, Kun Wang, Mojie Cheng, and Zhigang Shao. "High-performance solid oxide electrolysis cell with the ordered straight pores in the support." Journal of Power Sources 591 (2024): 233868.

[11] Zhao, Zhe, Shuai Tang, Zhongbo Liu, Longsheng Cao, Mojie Cheng, and Zhigang Shao. "Efficient and stable heterostructured air electrode for solid oxide steam electrolysis." International Journal of Hydrogen Energy 48, no. 15 (2023): 5764-5773.

[12] Wolf, Stephanie E., Vaibhav Vibhu, Eric Tröster, Izaak C. Vinke, Rüdiger-A. Eichel, and L. G. J. De Haart. "Steam electrolysis vs. co-electrolysis: Mechanistic studies of long-term solid oxide electrolysis cells." Energies 15, no. 15 (2022): 5449.

[13] Li, Yitong, Yunfeng Tian, Jin Li, Jian Pu, and Bo Chi. "Sr-free orthorhombic perovskite Pr0. 8Ca0. 2Fe0. 8Co0. 2O3-δ as a high-performance air electrode for reversible solid oxide cell." Journal of Power Sources 528 (2022): 231202.

[14] Wang, Wenjie, Yunfeng Tian, Yun Liu, Nalluri Abhishek, Yitong Li, Bo Chi, and Jian Pu. "Tailored Sr-Co-free perovskite oxide as an air electrode for high-performance reversible solid oxide cells." Science China Materials 64, no. 7 (2021): 1621-1631.

[15] Zhao, Zhe, Xiuling Wang, Shuai Tang, Mojie Cheng, and Zhigang Shao. "High-performance oxygen electrode Ce0. 9Co0. 1O2-δ-LSM-YSZ for hydrogen production by solid oxide electrolysis cells." International Journal of Hydrogen Energy 46, no. 50 (2021): 25332-25340.

[16] Kukk, Freddy, Priit Möller, Rait Kanarbik, and Gunnar Nurk. "Study of long-term stability of Ni-Zr0. 92Y0. 08O2-δ| Zr0. 92Y0. 08O2-δ| Ce0. 9Gd0. 1O2-δ| Pr0. 6Sr0. 4CoO3-δ at SOFC and SOEC mode." Energies 14, no. 4 (2021): 824.

[17] Kim, Sangcho, Dong Woo Joh, Dong-Young Lee, Jieun Lee, Hye Sung Kim, Muhammad Zubair Khan, Jong Eun Hong et al. "Microstructure tailoring of solid oxide electrolysis cell air electrode to boost performance and long-term durability." Chemical Engineering Journal 410 (2021): 128318.

[18] Schefold, Josef, Annabelle Brisse, Alexander Surrey, and Christian Walter. "80,000 current on/off cycles in a one year long steam electrolysis test with a solid oxide cell." International Journal of Hydrogen Energy 45, no. 8 (2020): 5143-5154.

[19] Lang, Michael, Sebastian Raab, Michelle Sophie Lemcke, Corinna Bohn, and Matthias Pysik. "Long-Term Behavior of a Solid Oxide Electrolyzer (SOEC) Stack▲." Fuel Cells 20, no. 6 (2020): 690-700.

[20] Bian, Liuzhen, Changyang Liu, Shuting Li, Jun Peng, Xiaowei Li, Lili Guan, Yuanyuan Liu, Ji Hua Peng, Shengli An, and Xiwen Song. "Highly stable La0. 5Sr0. 5Fe0. 9Mo0. 1O3-δ electrode for reversible symmetric solid oxide cells." International Journal of Hydrogen Energy 45, no. 38 (2020): 19813-19822.

[21] Shimada, Hiroyuki, Yoshinobu Fujimaki, and Yoshinobu Fujishiro. "Highly active and durable La0. 4Sr0. 6MnO3− δ and Ce0. 8Gd0. 2O1. 9 nanocomposite electrode for high-temperature reversible solid oxide electrochemical cells." Ceramics International 46, no. 11 (2020): 19617-19623.

[22] Tong, Xiaofeng, Simona Ovtar, Karen Brodersen, Peter Vang Hendriksen, and Ming Chen. "Large-area solid oxide cells with La0. 6Sr0. 4CoO3-δ infiltrated oxygen electrodes for electricity generation and hydrogen production." Journal of Power Sources 451 (2020): 227742.

[23] Zuo, Xiaodong, Zhiyi Chen, Chengzhi Guan, Kongfa Chen, Sanzhao Song, Guoping Xiao, Yuepeng Pang, and Jian-Qiang Wang. "Molten salt synthesis of high-performance, nanostructured La0. 6Sr0. 4FeO3− δ oxygen electrode of a reversible solid oxide cell." Materials 13, no. 10 (2020): 2267.

[24] Trini, Martina, Anne Hauch, S. De Angelis, X. Tong, P. Vang Hendriksen, and M. Chen. "Comparison of microstructural evolution of fuel electrodes in solid oxide fuel cells and electrolysis cells." Journal of Power Sources 450 (2020): 227599.

[25] Rabuni, Mohamad Fairus, Nattapat Vatcharasuwan, Tao Li, and Kang Li. "High performance micro-monolithic reversible solid oxide electrochemical reactor." Journal of Power Sources 458 (2020): 228026.

[26] Sun, X., P. V. Hendriksen, M. B. Mogensen, and M. Chen. "Degradation in solid oxide electrolysis cells during long term testing." Fuel Cells 19, no. 6 (2019): 740-747.

[27] Wang, Ruofan, Emir Dogdibegovic, Grace Y. Lau, and Michael C. Tucker. "Metal-supported solid oxide electrolysis cell with significantly enhanced catalysis." Energy Technology 7, no. 5 (2019): 1801154.

[28] Shimada, Hiroyuki, Toshiaki Yamaguchi, Haruo Kishimoto, Hirofumi Sumi, Yuki Yamaguchi, Katsuhiro Nomura, and Yoshinobu Fujishiro. "Nanocomposite electrodes for high current density over 3 A cm− 2 in solid oxide electrolysis cells." Nature communications 10, no. 1 (2019): 5432.

[29] Zhang, Shan-Lin, Hongqian Wang, Matthew Y. Lu, Cheng-Xin Li, Chang-Jiu Li, and Scott A. Barnett. "Electrochemical performance and stability of SrTi0. 3Fe0. 6Co0. 1O3-δ infiltrated La0. 8Sr0. 2MnO3Zr0. 92Y0. 16O2-δ oxygen electrodes for intermediate-temperature solid oxide electrochemical cells." Journal of Power Sources 426 (2019): 233-241.

[30] Fang, Qingping, Carolin E. Frey, Norbert H. Menzler, and Ludger Blum. "Electrochemical performance and preliminary post-mortem analysis of a solid oxide cell stack with 20,000 h of operation." Journal of The Electrochemical Society 165, no. 2 (2018): F38.

[31] Kim, Junyoung, Areum Jun, Ohhun Gwon, Seonyoung Yoo, Meilin Liu, Jeeyoung Shin, Tak-Hyoung Lim, and Guntae Kim. "Hybrid-solid oxide electrolysis cell: A new strategy for efficient hydrogen production." Nano Energy 44 (2018): 121-126.

[32] Yoon, Kyung Joong, Mridula Biswas, Hyo-Jin Kim, Mansoo Park, Jongsup Hong, Hyoungchul Kim, Ji-Won Son, Jong-Ho Lee, Byung-Kook Kim, and Hae-Weon Lee. "Nano-tailoring of infiltrated catalysts for high-temperature solid oxide regenerative fuel cells." Nano Energy 36 (2017): 9-20.

[33] Jun, Areum, Junyoung Kim, Jeeyoung Shin, and Guntae Kim. "Achieving high efficiency and eliminating degradation in solid oxide electrochemical cells using high oxygen-capacity perovskite." Angewandte Chemie International Edition 55, no. 40 (2016): 12512-12515.

[34] Myung, Jae-ha, Dragos Neagu, David N. Miller, and John TS Irvine. "Switching on electrocatalytic activity in solid oxide cells." Nature 537, no. 7621 (2016): 528-531.

[35] Tan, Yuan, Nanqi Duan, Ao Wang, Dong Yan, Bo Chi, Ning Wang, Jian Pu, and Jian Li. "Performance enhancement of solution impregnated nanostructured La0. 8Sr0. 2Co0. 8Ni0. 2O3-δ oxygen electrode for intermediate temperature solid oxide electrolysis cells." Journal of Power Sources 305 (2016): 168-174.

[36] Fang, Qingping, Ludger Blum, and Norbert H. Menzler. "Performance and degradation of solid oxide electrolysis cells in stack." Journal of The Electrochemical Society 162, no. 8 (2015): F907.

[37] Schefold, Josef, Annabelle Brisse, and Hendrik Poepke. "Long-term steam electrolysis with electrolyte-supported solid oxide cells." Electrochimica Acta 179 (2015): 161-168.

[38] Mahmood, Asif, Saira Bano, Ji Haeng Yu, and Kew-Ho Lee. "High-performance solid oxide electrolysis cell based on ScSZ/GDC (scandia-stabilized zirconia/gadolinium-doped ceria) bi-layered electrolyte and LSCF (lanthanum strontium cobalt ferrite) oxygen electrode." Energy 90 (2015): 344-350.

[39] Hong, Jongsup, Hyo-Jin Kim, Sun-Young Park, Jin-Ho Lee, Su-Byung Park, Jong-Ho Lee, Byung-Kook Kim, Hae-Joon Je, Jae Yuk Kim, and Kyung Joong Yoon. "Electrochemical performance and long-term durability of a 200 W-class solid oxide regenerative fuel cell stack." International journal of hydrogen energy 39, no. 35 (2014): 20819-20828.

[40] Fan, Hui, Michael Keane, Na Li, Dan Tang, Prabhakar Singh, and Minfang Han. "Electrochemical stability of La0. 6Sr0. 4Co0. 2Fe0. 8O3− δ-infiltrated YSZ oxygen electrode for reversible solid oxide fuel cells." International Journal of Hydrogen Energy 39, no. 26 (2014): 14071-14078.

[41] Lee, Sung-il, Jeonghee Kim, Ji-Won Son, Jong-Ho Lee, Byung-Kook Kim, Hae-June Je, Hae-Weon Lee, Huesup Song, and Kyung Joong Yoon. "High performance air electrode for solid oxide regenerative fuel cells fabricated by infiltration of nano-catalysts." Journal of Power Sources 250 (2014): 15-20.

[42] Zhang, Xiaoyu, James E. O'Brien, Robert C. O'Brien, Joseph J. Hartvigsen, Greg Tao, and Gregory K. Housley. "Improved durability of SOEC stacks for high temperature electrolysis." International Journal of Hydrogen Energy 38, no. 1 (2013): 20-28.

[43] Jensen, Søren Højgaard, Xiufu Sun, Sune Dalgaard Ebbesen, Ruth Knibbe, and Mogens Mogensen. "Hydrogen and synthetic fuel production using pressurized solid oxide electrolysis cells." International journal of hydrogen energy 35, no. 18 (2010): 9544-9549.
